# Examining neuroimaging biomarkers, plasma biomarkers and cognitive functions in patients with recovered COVID-19 infection: a multicentre study using 7T MRI

**DOI:** 10.1093/braincomms/fcag045

**Published:** 2026-03-09

**Authors:** Jr-Jiun Liou, Tales Santini, Jinghang Li, Monica Gireud-Goss, Tiffany F Kautz, Julie Parker-Garza, Juan Carlos Guerrero, Vibhuti Patel, Oluwatobi F Adeyemi, Gabriel A de Erausquin, Valentina R Garbarino, Mohamad Habes, Jayandra J Himali, Christof Karmonik, Beth E Snitz, Joseph M Mettenburg, Minjie Wu, Howard J Aizenstein, Anna L Marsland, Peter J Gianaros, Richard Bowtell, Olivier Mougin, Penny A Gowland, Mohammad Zia Katshu, Farhaan S Vahidy, Timothy D Girard, Heidi I L Jacobs, Akram A Hosseini, Sudha Seshadri, Tamer S Ibrahim, 7T MRI COVID Consortium, Sudha Seshadri, Farhaan S Vahidy, Timothy D Girard, Heidi I L Jacobs, Akram A Hosseini, Tamer S Ibrahim

**Affiliations:** University of Pittsburgh, Pittsburgh, PA 15260, USA; University of Pittsburgh, Pittsburgh, PA 15260, USA; University of Pittsburgh, Pittsburgh, PA 15260, USA; University of Texas Health Science Center at San Antonio, San Antonio, TX 78229, USA; University of Texas Health Science Center at San Antonio, San Antonio, TX 78229, USA; University of Texas Health Science Center at San Antonio, San Antonio, TX 78229, USA; University of Texas Health Science Center at San Antonio, San Antonio, TX 78229, USA; University of Texas Health Science Center at San Antonio, San Antonio, TX 78229, USA; University of Nottingham, Nottingham NG7 2RD, UK; University of Texas Health Science Center at San Antonio, San Antonio, TX 78229, USA; University of Texas Health Science Center at San Antonio, San Antonio, TX 78229, USA; University of Texas Health Science Center at San Antonio, San Antonio, TX 78229, USA; University of Texas Health Science Center at San Antonio, San Antonio, TX 78229, USA; Houston Methodist Research Institute, Houston, TX 77030, USA; University of Pittsburgh, Pittsburgh, PA 15260, USA; University of Pittsburgh, Pittsburgh, PA 15260, USA; University of Pittsburgh, Pittsburgh, PA 15260, USA; University of Pittsburgh, Pittsburgh, PA 15260, USA; University of Pittsburgh, Pittsburgh, PA 15260, USA; University of Pittsburgh, Pittsburgh, PA 15260, USA; University of Nottingham, Nottingham NG7 2RD, UK; University of Nottingham, Nottingham NG7 2RD, UK; University of Nottingham, Nottingham NG7 2RD, UK; University of Nottingham, Nottingham NG7 2RD, UK; Houston Methodist Research Institute, Houston, TX 77030, USA; University of Pittsburgh, Pittsburgh, PA 15260, USA; Massachusetts General Hospital, Harvard University, Boston, MA 02114, USA; University of Nottingham, Nottingham NG7 2RD, UK; University of Texas Health Science Center at San Antonio, San Antonio, TX 78229, USA; University of Pittsburgh, Pittsburgh, PA 15260, USA

**Keywords:** hospitalization, white matter hyperintensity, hippocampal subfield, glial fibrillary acidic protein, memory

## Abstract

We examined the impact of COVID-19 hospitalization on neuroimaging biomarkers and the association of these neuroimaging biomarkers with cognitive measures and plasma biomarkers. A total of 179 dementia-free people, including 52 hospitalized COVID-19 patients, across four medical centres in the USA and UK underwent 7T brain MRI scans, cognitive tests and blood collection. We found that hospitalized patients exhibited a comparable white matter hyperintensity burden, lower total hippocampal volume and lower plasma glial fibrillary acidic protein concentration, along with poorer memory performance, compared to age-matched non-hospitalized participants. Higher white matter hyperintensity burden was associated with older age, worse cognitive scores and higher plasma biomarker levels; higher total hippocampal volume was associated with younger age, better cognitive scores and lower plasma phosphorylated tau levels. However, these correlation coefficients did not differ between the hospitalized and non-hospitalized groups. Longitudinal studies are needed to clarify the long-term impact of COVID-19-related hospitalization.

## Teaser

Liou *et al*. found hospitalized COVID-19 patients had lower hippocampal volume, reduced plasma glial fibrillary acidic protein and poorer memory than age-matched controls, despite similar white matter hyperintensity burden. Biomarker–cognition correlations aligned with age and did not differ by hospitalization status. Longitudinal studies need to clarify the long-term impacts of COVID-19-related hospitalization.

## Graphical Abstract

**Graphical Abstract:**
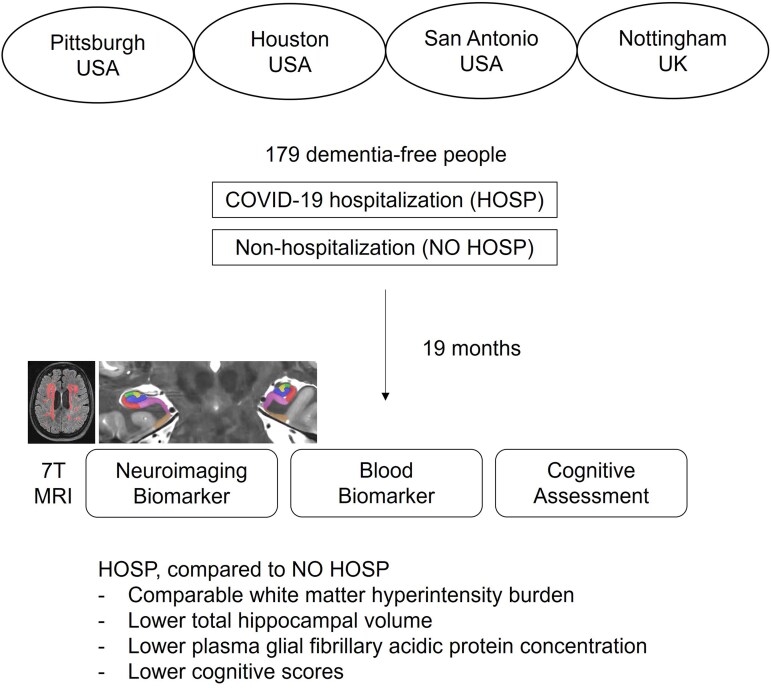

## Introduction

Structural imaging of the brain using magnetic resonance imaging (MRI) has revealed a wide range of abnormal findings after COVID-19 infection in acute settings.^1-5^ One recent longitudinal study found that people with clinically overt COVID-19 infection showed greater brain atrophy and cortical thinning as well as increased cognitive decline compared to controls who never developed an acute COVID-19 infection.^6^ Among those with acute infection, older age, female sex and hospitalizations have been associated with a higher risk for post-acute sequelae of COVID-19 illness also known as long COVID-19.^7^ Severe COVID-19 illness culminating in hospitalization has been associated with memory deficits^8^ and multiorgan dysfunction.^9^ These patients commonly exhibit different brain morphometrics that can be visualized using MRI, such as increased white matter hyperintensity (WMH) burden^9^ and higher grey matter volumes in the olfactory cortices^10^ compared to non-hospitalized controls. A recent study found that hospitalized patients exhibited increased volumes in the subiculum, a brain region in the medial temporal lobe, compared to non-hospitalized patients,^11^ as well as higher levels of plasma markers of neurological damage and neurodegeneration including neurofilament light (NfL) and glial fibrillary acidic protein (GFAP) compared to normative matched controls.^12^ Among hospitalized COVID-19 patients without a history of dementia, plasma total tau, phosphorylated tau 181, GFAP and NfL were significantly higher in those who did not survive in the hospital compared to those who survived.^13^ This study also found that NfL and GFAP were higher in COVID-19 patients than in non-COVID-19 controls with normal cognition, mild cognitive impairment or Alzheimer’s disease. Population studies found COVID-19 viral infection associated with new-onset Alzheimer’s disease at 12 months, especially in those above 80 years and in females.^14-18^

High-resolution and high-contrast 7 Tesla (7T) MRI can detect subtle injuries in the brain, with studies showing higher detection rates of white matter lesions,^19^ and cerebral microinfarcts,^20^ as well as specific and non-specific white matter hyperintensities,^21^ perivascular spaces,^22^ and volumetrics in hippocampal subfields.^23,24^ Most recently, a comprehensive study compared paired 7T and 3T brain morphometrics with age among 350 healthy participants and demonstrated that 7T improves statistical power and reduces required sample size.^25^

We aimed to investigate the impact of COVID-19 hospitalization on neuroimaging biomarkers and the association of these neuroimaging biomarkers with cognitive measures and plasma biomarkers within a diverse multinational cohort recruited as part of the 7T MRI COVID Consortium.

## Materials and methods

### 7T MRI COVID Consortium

With Institutional Review Board approvals from the University of Pittsburgh, Houston Methodist Research Institute, the University of Texas Health San Antonio and the University of Nottingham, participants were identified either from outreach referrals, clinical referrals or inpatient hospital records at four locations: Pittsburgh, USA; Houston, USA; San Antonio, USA; and Nottingham, UK. Institutional Review Board approval and informed consent were obtained during enrolment into the study by the participants according to the Declaration of Helsinki. Participants were screened and enrolled between May 2020 and October 2022. Prior to their initial visit, participants underwent a comprehensive informed consent process. To study the effects of COVID-19 infection in older adults without evidence of cognitive impairment, eligible participants were between the ages of 45 and 85 and had no pre-existing dementia, as screened using the Informant Questionnaire on Cognitive Decline in the Elderly^26^ and the Clinical Dementia Rating scale.^27^ Participants were asked to participate in two visits, the first to collect medical history, cognitive assessment and a peripheral blood sample and the second to complete a 75 min 7T MRI scan. A total of 179 participants enrolled and completed 7T MRI scans (Supplementary Fig. 1).

### COVID-19 severity

Participants who were hospitalized for COVID-19 (HOSP) and those who were not hospitalized (NO HOSP, which included mild and not-reported COVID-19 infection in the last 6–24 months prior to 7T MRI visit) were recruited and imaged. According to the WHO Clinical Classification,^28^ the severity of acute COVID-19 illness can be categorized as mild, moderate, severe or critical (Supplementary Table 1).

### Demographic information and medical history

Demographic assessments included age, biological sex, race and ethnicity, as well as occupational history utilizing a PhenX toolkit^29^ and information on current and former smoking, alcohol and recreational drug use. Medical history was reported by the participants, including cardiovascular and neurological conditions. The presence of diabetes, hypertension, hypercholesterolaemia, cancer, B12 deficiency, thyroid disease, sleep apnoea, obesity (BMI > 30), atrial fibrillation, congestive heart failure, heart valve replacement or repair, migraine and head injury were recorded. The number of COVID-19 and influenza vaccination doses was also recorded.

### Cognitive, mood and other neuropsychological assessments

Comprehensive evaluations encompassing cognitive, neuropsychological, mood and functional domains were conducted. The assessment battery comprised the Uniform Data Set 3 (UDS3) neuropsychological test battery from the National Alzheimer’s Coordinating Center (NACC) and NIH Toolbox. The cognitive test battery included Montreal cognitive assessment (MoCA^30^) and assessed verbal memory (Craft immediate/delayed recall^31^), visual memory (Benson figure recall^32^), psychomotor speed and cognitive flexibility (Trail making test A and B^33^) and visuospatial construction (Benson figure copy^32^).

### Plasma biomarkers

Peripheral blood was collected using established phlebotomy practices^34,35^ into an EDTA phlebotomy tube and centrifuged at 2000 × g for 10 min at 4°C. The subsequent plasma was aliquoted into polypropylene tubes and stored frozen at −80°C. Frozen samples were shipped to the Glenn Biggs Institute for Alzheimer’s and Neurodegenerative Diseases in San Antonio, TX, USA, for biomarker analysis. Plasma amyloid beta 40 (Ab40), amyloid beta 42 (Ab42), glial fibrillary acidic protein (GFAP), neurofilament light (NfL) and phosphorylated tau 181 (pTau181) were measured using the Simoa Neurology 4-Plex E and Simoa p-Tau 181 v2.1 assays manufactured by Quanterix (Billerica, USA).

### MRI acquisition

Participants in Pittsburgh^36^ were imaged using a 7T Siemens MAGNETOM scanner (Erlangen, Germany) equipped with the radiofrequency Tic-Tac-Toe head coil^37,38^ at the University of Pittsburgh. Participants in Houston and San Antonio^39^ were imaged using a 7T Siemens MAGNETOM Terra (Erlangen, Germany) at the Houston Methodist Hospital Research Institute with a commercial head coil manufactured by Nova Medical (Wilmington, USA) whereas participants in Nottingham^40^ were imaged using a 7T Philips Achieva (Amsterdam, Netherlands), also using the commercial head coil manufactured by Nova Medical (Wilmington, USA). The sample sizes of HOSP and NO HOSP at each site are provided in Supplementary Table 2. Each MRI scan included 0.55 mm isotropic T1-MP2RAGE, 0.75 × 0.75 × 1.5 mm T2-FLAIR and 0.375 × 0.375 × 1.5 mm T2-TSE. T2-TSE images were acquired perpendicular to the main axis of the hippocampus for segmentation of hippocampal subfields. Comprehensive sequence parameters were provided in Supplementary Table 3. The MRI scans were performed at 19.0 ± 7.5 months after hospital discharge in the HOSP group.

### MRI harmonization, segmentation and quality control

Intracranial volume (ICV) estimation, WMH segmentation and hippocampal subfield segmentation from T1-MP2RAGE, T2-FLAIR and T2-TSE were performed. ICVs were estimated from T1-MP2RAGE using SynthStrip (https://github.com/nipreps/synthstrip),^41^ which leverages deep learning techniques for improved accuracy. Precise estimation of ICV is crucial for normalizing subregional volumes to account for individual head size variability.

Segmentation of WMH from the T2-FLAIR images was performed using our in-house deep learning-based model wmh_seg2d.^42^ The scripts were shared via GitHub (https://github.com/jinghangli98/wmh_seg). This model, which was trained in a diverse dataset of 1.5, 3 and 7T MRI and using data augmentation for the common artefacts seen in 7T MRI, was utilized in the T2-FLAIR images and provides a more accurate WMH segmentation compared to FreeSurfer segmentation of T1 images.^42^ The data augmentation, which included the addition of inhomogeneity and noise, was implemented using TorchIO,^43^ serving as a harmonization step for multicentre imaging data. All WMH segmentations underwent visual inspection using ITK-SNAP^44^ before proceeding to volume extraction.

For segmentation of the hippocampal subfields, we first removed the characteristic background noise from the T1-MP2RAGE by combining the white matter, grey matter, cerebrospinal fluid and skull segmentations from the second inversion of the T1-MP2RAGE sequence obtained using Statistical Parametric Mapping (SPM) 12 package (https://github.com/spm/spm12)^45^ and masking the T1-MP2RAGE. We performed an image harmonization between our multicentre imaging data and the atlas in two steps. First, the T2-TSE images were denoised using a variance stabilizing transformation (VST) (https://github.com/yu-lab-vt/ConvexVST) and the block-matching and 4D filtering algorithm,^23,46^ and the resultant image was bias corrected using SPM12. Second, we denoised the T2-TSE images from the IKND Magdeburg Young Adult 7T^47^ atlas using the same strategy as applied to our T2-TSE images. We then estimated the volumes of hippocampal subfields using the Automatic Segmentation of Hippocampal Subfields (ASHS) package (https://github.com/pyushkevich/ashs).^48^ The hippocampal subfield segmentations were then visually inspected and corrected following the Berron'17 protocol.^47^ In the main analysis, we combined the cornu Ammonis (CA) 2, CA3 and dentate gyrus (DG) subfields due to the characteristic small volumes of the CA2 and CA3 subfields and, therefore, potentially higher variability of labelling. Neuroimages that were harmonized and segmented but did not pass quality control for either WMH or hippocampal subfields due to excessive motion artefacts and poor delineation were excluded (*n* = 16) from the subsequent analysis. A total of 163 were included for subsequent statistical analysis.

### Statistical analysis

The total WMH volume, total hippocampal volume, and hippocampal subfield volume were adjusted using age, sex and ICV as the covariates to account for subject variability using the Multiple Linear Regression function in MathWorks MATLAB (version R2023a). All other comparisons were conducted using GraphPad Prism (version 10.2.0.392).

To compare the means of the HOSP and NO HOSP groups, unpaired *t*-tests were employed; the difference between means and the 95% confidence interval (CI) were reported. The resulting *P*-values were corrected for multiple comparisons with the 10% false discovery rate (FDR), and the corrected *P*-values (or *q*-values) are provided.

To assess the association of neuroimaging biomarkers with age, cognitive performance or plasma biomarkers, first Kolmogorov–Smirnov, Shapiro–Wilk, Anderson–Darling and D’Agostino–Pearson normality tests were performed to assess whether values were normally distributed. If three of the four tests indicated normality, subsequent Pearson correlations were employed to assess associations in the HOSP and NO HOSP groups separately; if not normal, Spearman non-parametric correlation was performed to assess association. Correlation coefficient *r* with 95% CI was reported along with the corresponding *P*-value.

To test whether the correlation coefficients in the HOSP and NO HOSP groups differed significantly, Fisher’s *r*-to-*z* transformation was applied, and the resulting *z*-scores were compared. A significant difference between two correlation coefficients was defined as an absolute *z*-score >1.96. For cases where the correlation slopes intersected, the data were likely skewed due to outliers, leading to a definitive difference between slopes. Therefore, the Fisher *r*-to-*z* transformation was not performed, and these cases were labelled as not applicable (na) in the table.

## Results

### Comparison of COVID-19 severity

In the HOSP group (*n* = 52), the average duration of hospitalization or length of stay (LOS) was 12.3 ± 14.7 (range 1–76) days (Table 1). Eighteen of the 52 participants were in an intensive care unit (ICU) for 9.9 ± 7.4 (range 2–28) days. According to the WHO classification of COVID-19 severity, HOSP participants included 27% critical, 54% severe, 13% moderate and 6% mild cases; the severity was significantly associated with the length of stay (*P* = 0.0003) (Supplementary Fig. 2). In contrast, the NO HOSP participants included 3% severe, 6% moderate and 38% mild, and the remaining 53% were asymptomatic. Three of the 111 NO HOSP participants reported receiving oxygen via nasal cannula or face mask and were therefore included in the Severe category; 7 in the NO HOSP group showed clinical signs of non-severe pneumonia, considered as ‘moderate’. When comparing the HOSP and NO HOSP groups, the average disease severity in HOSP was significantly higher than that of NO HOSP (*P* < 0.0001). No difference between HOSP and NO HOSP was detected in the percentage of participants who received COVID-19 vaccines, the number of COVID-19 vaccine doses received or percentage of people who received one or more influenza vaccines.

**Table 1:** Comparison of COVID-19 severity, demographic information and medical history of 163 participants with (HOSP) or without hospitalization (NO HOSP)

	HOSP *n* = 52 No. (%)	NO HOSP *n* = 111 No. (%)	*P*-value
COVID-19			
COVID-19 severity			
Critical, No. (%); mean LOS	14 (27); 23.8	0	**<0**.**0001**
Severe, No. (%); mean LOS	28 (54); 10.7	3 (3)^a^	
Moderate, No. (%); mean LOS	7 (13); 1.7	7 (6)^b^	
Mild, No. (%); mean LOS	3 (6); 1.7	42 (38)	
No reported symptoms, No. (%)	0	59 (53)	
Received COVID-19 vaccine, No. (%)	44 (85)	76 (86)	0.23
COVID-19 vaccine doses, mean (SD)	3.0 (0.8)	3.0 (0.6)	0.76
Received influenza vaccine, No. (%)	31 (60)	52 (59)	0.66
Demographic information			
Age, mean (SD)	61.1 (7.4)	61.5 (8.4)	0.74
Sex female, No. (%)	19 (37)	66 (59)	**0**.**0033**
Race White, No. (%)	36 (69)	89 (80)	0.06
Ethnicity Hispanic/Latino, No. (%)	7 (13)	19 (17)	0.53
Years of education, mean (SD)	14.8 (2.7)	15.8 (2.4)	**0**.**0205**
Medical history			
Diabetes, No. (%)	17 (33)	10 (11)^c^	**0**.**0021**
Hypertension, No. (%)	23 (44)	30 (34)^c^	0.28
Hypercholesterolaemia, No. (%)	22 (42)	32 (37)^c^	0.38
Cancer, No. (%)	8 (15)	15 (17)^c^	0.71
B12 deficiency, No. (%)	7 (13)	13 (15)^c^	0.83
Thyroid disease, No. (%)	4 (8)	17 (20)^c^	**0**.**0496**
Sleep apnoea, No. (%)	13 (25)	9 (10)^c^	**0**.**0191**
Obesity (BMI > 30), No. (%)	19 (37)	16 (18)^c^	**0**.**0174**
Atrial fibrillation, No. (%)	3 (6)	3 (3)^c^	0.55
Congestive heart failure, No. (%)	1 (2)	1 (1)^c^	0.73
Heart valve surgery, No. (%)	1 (2)	0^c^	0.20
Migraines, No. (%)	8 (15)	21 (24)^c^	0.20
Head injury, No. (%)	5 (10)	16 (18)^c^	0.14

The *t*-test *P*-values are reported. Cross-sectional comparisons reveal more severe COVID-19 symptoms, lower percentage of females, fewer years of education and higher percentage of diabetes, thyroid disease, sleep apnoea and obesity in the HOSP group. Statistically significant *P*-values are in bold. Abbreviations: BMI, body mass index; LOS, length of stay (days). ^a^Participants received oxygen via nasal cannula or face mask. ^b^Participants showed clinical signs of non-severe pneumonia. ^c^Data from 88 participants in the NO HOSP group.

### Comparison of demographics and medical history

There was no significant difference between HOSP and NO HOSP participants in age [difference between means (95% CI) = 0.45 (−2.25, 3.16) years], percentage White or percentage Hispanic/Latino (Table 1). The HOSP group had a lower percentage of females (37% versus 59%, *P* = 0.0033) and fewer years of education [difference between means (95% CI) = 0.98 (0.15, 1.81) years, *P* = 0.0205] than the NO HOSP group. Comorbidities, such as diabetes (33% versus 11%, *P* = 0.0021), sleep apnoea (25% versus 10%, *P* = 0.0191) and obesity (37% versus 18%, *P* = 0.0174), were more common whereas thyroid disease (8% versus 20%, *P* = 0.0496) was less common in the HOSP group than the NO HOSP group. No group differences were detected in hypertension, hypercholesterolaemia, cancer, B12 deficiency, atrial fibrillation, congestive heart failure, heart valve replacement or repair, migraine or head injury.

### Comparison of cognition and plasma biomarkers

HOSP participants had lower MoCA scores (24.9 versus 26.4, *P* = 0.0084, *q* = 0.0046), required more time to complete Trail making test B (97.6 versus 79.4, *P* = 0.0285, *q* = 0.0125) and had lower Craft immediate (12.6 versus 16.4, *P* < 0.0001, *q* = 0.0001) and delayed (12.0 versus 16.1, *P* = 0.0001, *q* = 0.0001) recall scores and lower Benson figure copy scores (15.2 versus 16.1, *P* = 0.0078, *q* = 0.0046) than NO HOSP participants (Table 2). No difference was detected in Trail making test A or Benson figure recall scores. The concentration of plasma GFAP was lower in the HOSP group than the NO HOSP group (73.1 versus 97.8 pg/ml, *P* = 0.0051, *q* = 0.0224). No group differences were detected in levels of any other plasma markers including NfL, Ab42, Ab40 and pTau181.

**Table 2:** Comparison of cognitive assessment, plasma biomarkers and MRI neuroimaging biomarkers of 163 participants with (HOSP) or without hospitalization (NO HOSP)

	HOSP *n* = 52 mean (SD)	NO HOSP *n* = 111 mean (SD)	Difference between means (95% CI)	*P*-value	*q*-value
Cognitive assessment					
MoCA	24.9 (3.7)	26.4 (2.9)	1.47 (0.38, 2.55)	**0.0084**	**0**.**0046**
Trail making test A	35.0 (22.5)	32.7 (14.3)	−2.29 (−8.24, 3.65)	0.45	0.1414
Trail making test B	97.6 (46.0)	79.4 (48.3)	−18.26 (−34.56, −1.95)	**0**.**0285**	**0**.**0125**
Craft immediate recall	12.6 (6.4)	16.4 (3.2)^a^	3.76 (2.09, 5.44)	**<0**.**0001**	**0**.**0001**
Craft delayed recall	12.0 (6.6)	15.6 (3.7)^a^	3.58 (1.77, 5.39)	**0**.**0001**	**0**.**0001**
Benson figure copy	15.2 (2.7)	16.1 (1.0)^a^	0.89 (0.24, 1.55)	**0**.**0078**	**0**.**0046**
Benson figure recall	12.1 (3.6)	12.6 (2.4)^a^	0.53 (−0.52, 1.58)	0.32	0.1173
Plasma biomarker (pg/ml)					
NfL	16.0 (8.1)	16.5 (7.5)^a^	0.46 (−2.29, 3.22)	0.74	0.6688
Ab42	6.8 (2.2)	6.7 (1.6)^a^	−0.11 (−0.79, 0.58)	0.76	0.6688
Ab40	101.2 (24.8)	106.6 (21.1)^a^	5.41 (−2.69, 13.52)	0.19	0.2787
GFAP	73.1 (39.7)	97.8 (51.6)^a^	24.74 (7.58, 41.90)	**0**.**0051**	**0**.**0224**
pTau181	20.9 (11.5)	18.6 (7.5)^a^	−2.27 (−5.57, 1.03)	0.18	0.2787
White matter hyperintensity					
WMHV/ICV	0.0029 (0.0056)	0.0021 (0.0021)	−0.0008 (−0.0020, 0.0004)	0.21	0.2310
Whole hippocampus (mm^3^)					
Left + right hippocampi	11 856 (1088)	12 227 (1070)	370 (0.42, 738.9)	**0**.**0497**	**0**.**0547**
Hippocampal subfields (mm^3^)					
Left CA1	718 (101)	745 (116)	35.18 (−2.71, 73.07)	0.07	0.2053
Left CA2 + CA3 + DG	635 (94)	658 (90)	29.34 (−2.24, 60.92)	0.07	0.2053
Left SUB	1073 (138)	1077 (121)	1.53 (−41.51, 44.56)	0.94	1.0000
Left ERC	869 (134)	873 (127)	0.78 (−42.36, 43.93)	0.97	1.0000
Right CA1	755 (98)	794 (113)	36.35 (0.81, 71.89)	**0**.**0450**	0.2053
Right CA2 + CA3 + DG	701 (101)	722 (87)	24.21 (−5.84, 54.26)	0.11	0.2420
Right SUB	1090 (148)	1111 (124)	12.07 (−31.21, 55.35)	0.58	1.0000
Right ERC	876 (122)	876 (138)	−3.85 (−48.13, 40.44)	0.86	1.0000

All rows are shown as mean (SD) along with difference between means (95% CI) and *t*-test *P*-values as well as the *q*-values after correcting for multiple comparisons by controlling the 10% false discovery rate (FDR). Cross-sectional comparisons reveal worse cognitive scores, lower plasma GFAP levels and lower total hippocampal volume in the HOSP group. Statistically significant *P*-values and *q*-values are in bold. Abbreviations: Ab40, amyloid beta 40; Ab42, amyloid beta 42; CA1, cornu Ammonis 1; CA2 + CA3 + DG, cornu Ammonis 2 + cornu Ammonis 3 + dentate gyrus; ERC, entorhinal cortex; GFAP, glial fibrillary acidic protein; ICV, intracranial volume; MoCA, Montreal cognitive assessment; NfL, neurofilament light; pTau181, phosphorylated tau 181; SUB, subiculum; WMHV, white matter hyperintensity volume. ^a^Data from 88 participants in the NO HOSP group.

### Comparison of white matter hyperintensity volume and its association with cognition and plasma biomarkers

**Figure 1:**
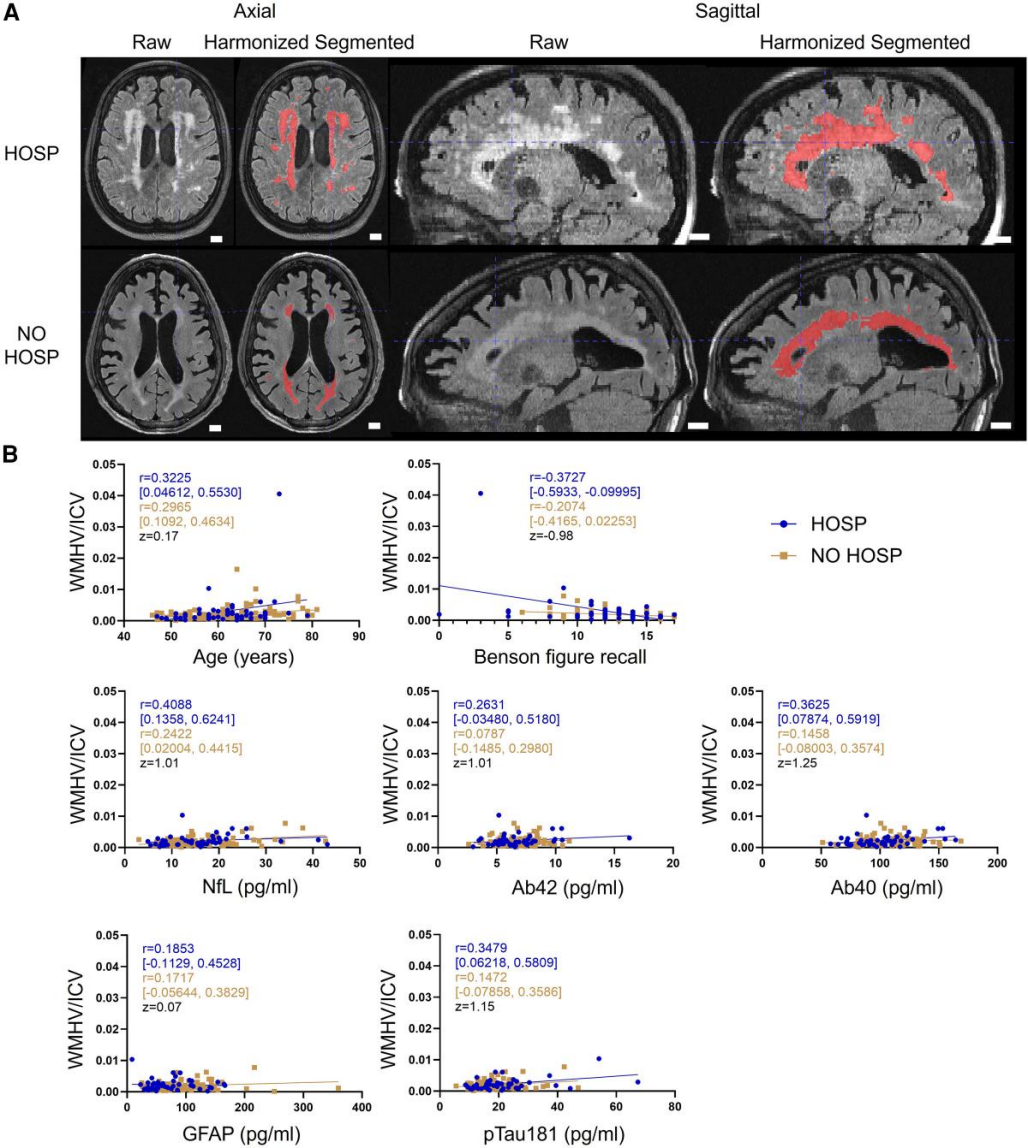
**White matter hyperintensity segmentation, quantification and association with cognitive performance and plasma biomarkers.** (**A**) Representative raw and harmonized + segmented T2-weighted fluid-attenuated inversion recovery (FLAIR) MRI images from two participants with the highest white matter hyperintensity (WMH) burden in each group in both axial and sagittal views. At the group level, no statistical difference is detected. Scale bar is 1.0 cm. (**B**) The association of WMH burden with age, Benson figure recall score or plasma biomarkers is comparable between hospitalized (HOSP, *n* = 52, circles) and non-hospitalized (NO HOSP, *n* = 88, squares) participants. Each datapoint represents a participant in each group. Spearman correlation coefficient *r* and 95% confidence interval in each group as well as the *z*-score comparing two correlation coefficients are reported. WMHV/ICV, white matter hyperintensity volume normalized by intracranial volume; NfL, neurofilament light; Ab42, amyloid beta 42; Ab40, amyloid beta 40; GFAP, glial fibrillary acidic protein; pTau181, phosphorylated tau 181.

Representative raw as well as harmonized and segmented T2-FLAIR images of the two participants with the highest WMH burden were presented in axial and sagittal views (Fig. 1A). At the group level, the HOSP and NO HOSP groups had comparable WMH burden (Table 2). In the HOSP group, total white matter hyperintensity volume (WMHV) was associated with older age (Spearman *r* = 0.3225, *P* = 0.0197), lower MoCA score (Spearman *r* = −0.3092, *P* = 0.0273), longer duration to complete Trail making test B (Spearman *r* = 0.3527, *P* = 0.0120), lower Benson figure copy (Spearman *r* = −0.4105, *P* = 0.0028)/recall (Spearman *r* = −0.3727, *P* = 0.0071) scores as well as increased plasma levels of NfL (Spearman *r* = 0.4088, *P* = 0.0035), Ab40 (Spearman *r* = 0.3625, *P* = 0.0113) and pTau181 (Spearman *r* = 0.3479, *P* = 0.0154) (Table 3). In the NO HOSP group, total WMH volume was only associated with older age (Spearman *r* = 0.2965, *P* = 0.0017) and increased plasma levels of NfL (Spearman *r* = 0.2422, *P* = 0.0284).

**Table 3:** Association of total white matter hyperintensity (WMH) volume with age, cognitive performance and plasma biomarkers of 163 participants with (HOSP) or without hospitalization (NO HOSP)

	Total WMH volume						
	HOSP			NO HOSP			*z*-score
	Spearman *r*	95% CI	*P*-value	Spearman *r*	95% CI	*P*-value	
Age	0.3225	0.04612 to 0.5530	**0.0197**	0.2965	0.1092 to 0.4634	**0**.**0017**	0.17
MoCA	−0.3092	−0.5448 to −0.02840	**0**.**0273**	0.1914	−0.01106 to 0.3788	0.06	na
Trail making test A	0.1084	−0.1804 to 0.3800	0.45	−0.0404	−0.2405 to 0.1630	0.69	na
Trail making test B	0.3527	0.07403 to 0.5803	**0**.**0120**	−0.0879	−0.2859 to 0.1173	0.39	na
Craft immediate recall	−0.2169	−0.4712 to 0.07073	0.13	0.0787	−0.1529 to 0.3022	0.49	na
Craft delayed recall	−0.1475	−0.4187 to 0.1479	0.31	0.0192	−0.2106 to 0.2470	0.87	na
Benson figure copy	−0.4105	−0.6215 to −0.1439	**0**.**0028**	0.0505	−0.1805 to 0.2762	0.66	na
Benson figure recall	−0.3727	−0.5933 to −0.09995	**0**.**0071**	−0.2074	−0.4165 to 0.02253	0.07	−0.98
NfL	0.4088	0.1358 to 0.6241	**0**.**0035**	0.2422	0.02004 to 0.4415	**0**.**0284**	1.01
Ab42	0.2631	−0.03480 to 0.5180	0.07	0.0787	−0.1485 to 0.2980	0.49	1.01
Ab40	0.3625	0.07874 to 0.5919	**0**.**0113**	0.1458	−0.08003 to 0.3574	0.19	1.25
GFAP	0.1853	−0.1129 to 0.4528	0.21	0.1717	−0.05644 to 0.3829	0.13	0.07
pTau181	0.3479	0.06218 to 0.5809	**0**.**0154**	0.1472	−0.07858 to 0.3586	0.19	1.15

Normality tests confirmed a non-Gaussian distribution; non-parametric Spearman correlation coefficient *r*, 95% confidence interval (CI) and *P*-value along with the *z*-score testing the difference between two correlation coefficients are reported. Correlation coefficients do not differ between HOSP and NO HOSP in age, cognitive performance or plasma biomarkers. Statistically significant *P*-values are in bold. Abbreviations: Ab40, amyloid beta 40; Ab42, amyloid beta 42; GFAP, glial fibrillary acidic protein; MoCA, Montreal cognitive assessment; na, not applicable; NfL, neurofilament light; pTau181, phosphorylated tau 181.

When comparing correlations between the two groups, the association of total WMH volume with age (Fisher *r*-to-*z* transformation *z* = 0.17), Benson figure recall (*z* = −0.98), plasma NfL (*z* = 1.01), plasma Ab42 (*z* = 1.01), plasma Ab40 (*z* = 1.25), plasma GFAP (*z* = 0.07) or plasma pTau181 (*z* = 1.15) in HOSP was not significantly different from that in NO HOSP given the absolute *z*-score <1.96 (Fig. 1B). Detailed scatter plots (Supplementary Fig. 3), along with the raw data (Supplementary Table 4), for WMH burden and its association with cognition and plasma biomarkers are provided in Supplementary material.

### Comparison of hippocampal volume and its association with cognition and plasma biomarkers

**Figure 2:**
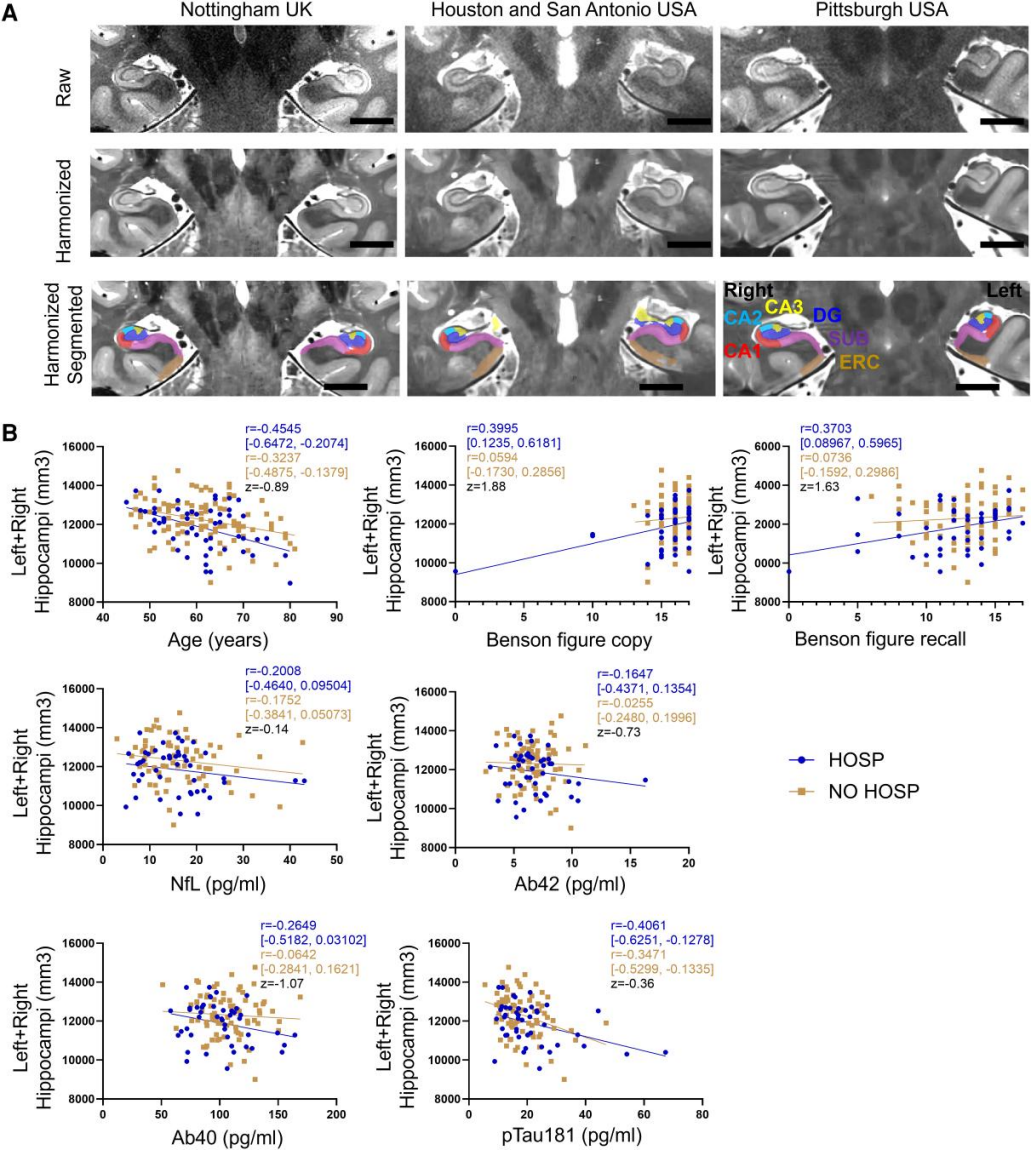
**Hippocampal subfield segmentation, quantification and association with cognitive performance and plasma biomarkers.** (**A**) Image harmonization and hippocampal subfield segmentation of T2-weighted turbo spin echo (TSE) MRI images are performed across three imaging sites. Scale bar is 1.0 cm. (**B**) The association of total hippocampal volume with age, Benson figure copy/recall scores or plasma biomarkers is comparable between hospitalized (HOSP, *n* = 52, circles) and non-hospitalized (NO HOSP, *n* = 88, squares) participants. Each datapoint represents a participant in each group. Pearson correlation coefficient *r* and 95% confidence interval in each group as well as the *z*-score comparing two correlation coefficients are reported. CA1, CA2, and CA3, cornu Ammonis 1, 2 and 3; DG, dentate gyrus; SUB, subiculum; ERC, entorhinal cortex; NfL, neurofilament light; Ab42, amyloid beta 42; Ab40, amyloid beta 40; pTau181, phosphorylated tau 181.

Representative raw, harmonized, and harmonized + segmented images of hippocampal subfields among the three imaging sites—Nottingham, UK; San Antonio, USA; and Pittsburgh, USA—are presented (Fig. 2A). The cornu Ammonis 1, 2, and 3 (CA1, CA2 and CA3), dentate gyrus (DG), subiculum (SUB) and entorhinal cortex (ERC) subfields are labelled in different colours. For quantitative comparisons, the left and right hemispheres were evaluated separately; the CA2, CA3 and DG were combined given the small volumes of each subfield and risk of mislabelling. The volume of left + right hippocampi [difference between means (95% CI) = 370 (0.42, 738.9) mm^3^, *P* = 0.0497, *q* = 0.0547, survived 10% FDR] was lower in the HOSP group than the NO HOSP group (Table 2). When examining the hippocampal subfields, the right CA1 [difference between means (95% CI) = 36.35 (0.81, 71.89) mm^3^, *P* = 0.0450, *q* = 0.2053] volume appeared to be lower in the HOSP group; however, the significance did not survive correction for multiple comparisons. In HOSP, the total hippocampal volume was associated with younger age (Pearson *r* = −0.4545, *P* = 0.0007), better MoCA (Pearson *r* = 0.3811, *P* = 0.0098), better Craft immediate recall (Pearson *r* = 0.5016, *P* = 0.0004), better Craft delayed recall (Pearson *r* = 0.4835, *P* = 0.0009), better Benson figure copy (Pearson *r* = 0.3995, *P* = 0.0060), better Benson figure recall (Pearson *r* = 0.3703, *P* = 0.0113) and lower plasma pTau18 level (Pearson *r* = −0.4061, *P* = 0.0056) (Table 4). In NO HOSP, the total hippocampal volume was only associated with younger age (Pearson *r* = −0.3237, *P* = 0.0009) and lower plasma pTau181 level (Pearson *r* = −0.3471, *P* = 0.0020).

**Table 4:** Association of total hippocampal volume with age, cognitive performance and plasma biomarkers of 163 participants with (HOSP) or without hospitalization (NO HOSP)

	Total hippocampal volume						
	HOSP			NO HOSP			*z*-score
	Pearson r	95% CI	*P*-value	Pearson r	95% CI	*P*-value	
Age	−0.4545	−0.6472 to −0.2074	**0.0007**	−0.3237	−0.4875 to −0.1379	**0**.**0009**	−0.89
MoCA	0.3811	0.09864 to 0.6068	**0**.**0098**	−0.0817	−0.2818 to 0.1252	0.44	na
Trail making test A	−0.2493	−0.5031 to 0.04424	0.09	0.0347	−0.1713 to 0.2378	0.74	na
Trail making test B	−0.1036	−0.3854 to 0.1959	0.50	0.0513	−0.1563 to 0.2546	0.63	na
Craft immediate recall	0.5016	0.2473 to 0.6912	**0**.**0004**	−0.1081	−0.3299 to 0.1251	0.36	na
Craft delayed recall	0.4835	0.2178 to 0.6824	**0**.**0009**	−0.1251	−0.3453 to 0.1080	0.29	na
Benson figure copy	0.3995	0.1235 to 0.6181	**0**.**0060**	0.0594	−0.1730 to 0.2856	0.62	1.88
Benson figure recall	0.3703	0.08967 to 0.5965	**0**.**0113**	0.0736	−0.1592 to 0.2986	0.54	1.63
NfL	−0.2008	−0.4640 to 0.09504	0.18	−0.1752	−0.3841 to 0.05073	0.13	−0.14
Ab42	−0.1647	−0.4371 to 0.1354	0.28	−0.0255	−0.2480 to 0.1996	0.83	−0.73
Ab40	−0.2649	−0.5182 to 0.03102	0.08	−0.0642	−0.2841 to 0.1621	0.58	−1.07
GFAP	0.0429	−0.2538 to 0.3323	0.78	−0.0432	−0.2676 to 0.1856	0.71	na
pTau181	−0.4061	−0.6251 to −0.1278	**0**.**0056**	−0.3471	−0.5299 to −0.1335	**0**.**0020**	−0.36

Normality tests confirmed a Gaussian distribution; Pearson correlation coefficient *r*, 95% confidence interval (CI) and *P*-value along with the *z*-score testing the difference between two correlation coefficients are reported. Correlation coefficients do not differ between HOSP and NO HOSP in age, cognitive performance or plasma biomarkers. Statistically significant *P*-values are in bold. Abbreviations: Ab40, amyloid beta 40; Ab42, amyloid beta 42; GFAP, glial fibrillary acidic protein; MoCA, Montreal cognitive assessment; na, not applicable; NfL, neurofilament light; pTau181, phosphorylated tau 181.

When comparing the two groups, the association of total hippocampal volume with age (Fisher *r*-to-*z* transformation *z* = −0.89), Benson figure copy score (*z* = 1.88), Benson figure recall score (*z* = 1.63), plasma NfL (*z* = −0.14), plasma Ab42 (*z* = −0.73), plasma Ab40 (*z* = −1.07) or plasma pTau181 (*z* = −0.36) in HOSP was not significantly different from that in NO HOSP given the absolute *z*-score <1.96 (Fig. 2B). Detailed scatter plots (Supplementary Fig. 4), along with the raw data (Supplementary Table 4), for total hippocampal volume and its association with cognition and plasma biomarkers are provided in Supplementary material.

To investigate which hippocampal subfields in the HOSP group were the primary contributors to the significant correlation of total hippocampal volume with age, cognitive performance and plasma pTau181, Pearson correlations were performed (Table 5). In HOSP, higher bilateral CA2 + CA3 + DG volumes correlated with younger age; higher bilateral SUB volumes correlated with higher MoCA score; higher bilateral CA2 + CA3 + DG, bilateral SUB and left ERC volumes correlated with higher Craft immediate/delayed recall scores; higher right CA2 + CA3 + DG and bilateral SUB volumes correlated with higher Benson figure copy/recall scores. Finally, higher volumes in bilateral CA1, right SUB and left ERC correlated with lower plasma pTau181 concentration.

**Table 5:** Association of hippocampal subfield volumes with age, cognitive performance and plasma pTau181 in the HOSP group

	HOSP					
	Pearson r	95% CI	*P*-value	Pearson r	95% CI	*P*-value
		Left CA1			Right CA1	
Age	−0.0847	−0.3470 to 0.1900	0.55	−0.2593	−0.4909 to 0.006397	0.06
MoCA	0.1754	−0.1210 to 0.4432	0.24	0.1405	−0.1529 to 0.4110	0.35
Craft immediate recall	0.1923	−0.1004 to 0.4543	0.20	0.0063	−0.2783 to 0.2899	0.97
Craft delayed recall	0.1674	−0.1327 to 0.4393	0.27	0.0111	−0.2801 to 0.3004	0.94
Benson figure copy	0.0625	−0.2287 to 0.3435	0.68	0.2227	−0.06558 to 0.4767	0.13
Benson figure recall	0.0879	−0.2044 to 0.3658	0.56	0.2719	−0.01330 to 0.5161	0.06
pTau181	−0.2935	−0.5380 to −0.003544	**0.0477**	−0.3167	−0.5559 to −0.02913	**0**.**0320**
	Left CA2 + CA3 + DG			Right CA2 + CA3 + DG		
Age	−0.4324	−0.6292 to −0.1836	**0**.**0012**	−0.4458	−0.6359 to −0.2047	**0**.**0006**
MoCA	0.2861	−0.004573 to 0.5322	0.05	0.1830	−0.1100 to 0.4467	0.22
Craft immediate recall	0.4684	0.2094 to 0.6660	**0**.**0009**	0.3230	0.04275 to 0.5561	**0**.**0252**
Craft delayed recall	0.4358	0.1631 to 0.6466	**0**.**0028**	0.3259	0.03928 to 0.5629	**0**.**0271**
Benson figure copy	0.1152	−0.1779 to 0.3895	0.44	0.2650	−0.02068 to 0.5107	0.07
Benson figure recall	0.2589	−0.03050 to 0.5083	0.08	0.3956	0.1256 to 0.6111	**0**.**0054**
pTau181	−0.2511	−0.5046 to 0.04228	0.09	−0.2594	−0.5112 to 0.03346	0.08
		Left SUB			Right SUB	
Age	−0.2637	−0.4985 to 0.007056	0.06	−0.3288	−0.5464 to −0.06954	**0**.**0143**
MoCA	0.3701	0.08947 to 0.5964	**0**.**0113**	0.4087	0.1377 to 0.6228	**0**.**0043**
Craft immediate recall	0.4770	0.2199 to 0.6721	**0**.**0007**	0.4947	0.2450 to 0.6829	**0**.**0004**
Craft delayed recall	0.4621	0.1950 to 0.6654	**0**.**0014**	0.4997	0.2449 to 0.6899	**0**.**0004**
Benson figure copy	0.3321	0.04965 to 0.5653	**0**.**0226**	0.4688	0.2131 to 0.6645	**0**.**0008**
Benson figure recall	0.2150	−0.07696 to 0.4729	0.15	0.3276	0.04796 to 0.5597	**0**.**0230**
pTau181	−0.1510	−0.4227 to 0.1457	0.32	−0.3726	−0.5982 to −0.09223	**0**.**0108**
		Left ERC			Right ERC	
Age	−0.1326	−0.3914 to 0.1455	0.35	−0.0721	−0.3335 to 0.1995	0.60
MoCA	0.1227	−0.1772 to 0.4018	0.42	0.1425	−0.1542 to 0.4156	0.34
Craft immediate recall	0.4587	0.1943 to 0.6610	**0**.**0013**	0.2554	−0.03433 to 0.5055	0.08
Craft delayed recall	0.4118	0.1309 to 0.6315	**0**.**0055**	0.2818	−0.01276 to 0.5314	0.06
Benson figure copy	0.2535	−0.03972 to 0.5065	0.09	0.2886	0.001615 to 0.5317	**0**.**0491**
Benson figure recall	0.2735	−0.01827 to 0.5223	0.07	0.1420	−0.1513 to 0.4124	0.34
pTau181	−0.4028	−0.6227 to −0.1239	**0**.**0061**	−0.2688	−0.5212 to 0.02687	0.07

Normality tests confirmed a Gaussian distribution; Pearson correlation coefficient *r*, 95% confidence interval (CI) and *P*-value were reported. In HOSP, higher bilateral CA2 + CA3 + DG volumes correlated with younger age; higher bilateral SUB volumes correlated with higher MoCA score; higher bilateral CA2 + CA3 + DG, bilateral SUB and left ERC volumes correlated with higher Craft immediate/delayed recall scores; higher right CA2 + CA3 + DG and bilateral SUB volumes correlated with higher Benson figure copy/recall scores. Finally, higher volumes in bilateral CA1, right SUB and left ERC correlated with lower plasma pTau181 concentration. Statistically significant *P*-values are in bold. Abbreviations: CA1, cornu Ammonis 1; CA2 + CA3 + DG, cornu Ammonis 2 + cornu Ammonis 3 + dentate gyrus; ERC, entorhinal cortex; MoCA, Montreal cognitive assessment; pTau181, phosphorylated tau 181; SUB, subiculum.

## Discussion

In this study, we investigated the impact of COVID-19 hospitalization on WMH and hippocampal volumes and their associations with cognition and plasma biomarkers within a diverse multinational cohort using 7T MRI. In the hospitalized group, we found comparable WMH burden, lower total hippocampal volume, lower plasma GFAP concentration and worse memory scores when compared to the age-matched non-hospitalized group.

WMH associations with COVID-19 are mixed in the literature. In large cohorts of hospitalized patients with COVID-19, 35%^49^ to 55%^50^ show non-specific but likely chronic WMH. When examined in detail, the reported chronic WMH is predominantly located within the subcortical and basal ganglia.^49^ It is possible that greater WMH accumulation may have resulted from severe COVID-19 infection, including complications such as acute ischaemic stroke, intracranial haemorrhage and hypoxic injury.^51^ However, other studies reported no difference in WMH load between non-hospitalized and hospitalized COVID-19 cases^52^ or between ICU and non-ICU COVID-19 patients.^53^ In line with these studies, we also observed comparable WMH burden between the hospitalized and the age-matched non-hospitalized groups. Total WMH volume was associated with older age and increased plasma level of NfL. However, Fisher’s *r*-to-*z* transformation revealed that the correlation coefficients did not differ between the two groups.

The impact of COVID-19 on hippocampal volume remains to be investigated. A few studies have reported enlarged bilateral hippocampi in COVID-19 patients^10,54,55^ and the volume of the bilateral hippocampus correlates with memory loss, while the left hippocampal volume is associated with smell loss.^10^ In contrast, other studies reported that hippocampal atrophy in COVID-19 patients, compared to healthy controls, is associated with altered microstructural integrity in white matter and functional connectivity^11^ as well as reduced cortical thickness in the left hippocampus.^56^ Hospitalized patients have shown elevated plasma levels of GFAP and NfL compared to non-hospitalized patients, with GFAP levels further associated with greater subiculum volume.^11^ Other studies have similarly reported increased plasma GFAP and NfL in hospitalized COVID-19 patients compared to non-COVID-19 participants with normal cognition, mild cognitive impairment or Alzheimer’s disease^13^ as well as compared to age-matched controls.^57^ In our study, HOSP participants had lower total hippocampal volumes when compared to NO HOSP participants. Within the HOSP group, higher total hippocampal volume was associated with younger age; better MoCA, Craft immediate/delayed recall and Benson figure copy/recall scores; and lower plasma pTau18 level, whereas within the NO HOSP group, higher total hippocampal volume was only associated with younger age and lower plasma pTau181 level. A comparison of the correlation coefficients between the HOSP and NO HOSP groups revealed no significant differences. The associations observed within the HOSP group may reflect lower variance in this group compared to the NO HOSP group. Increased variance in the NO HOSP group may stem from pre-existing comorbidities and differences in brain health prior to the pandemic. One study investigating the hippocampus identified potential pathological mechanisms of COVID-19-related brain damage, including blood–brain barrier disruption, elevated interleukin levels and impaired hippocampal neurogenesis.^58^ These COVID-19-related neuropathological processes may underlie the observed lower performance on cognitive tests of verbal memory, learning, working memory and executive function observed in patients with COVID-19.

In the HOSP group, we found that several hippocampal subfields likely contributed to the correlations between total hippocampal volume and age, cognitive performance and plasma pTau181 levels: bilateral CA2 + CA3 + DG for age, bilateral SUB for MoCA and Craft immediate/delayed recall scores and the left ERC for pTau181. These results suggest that hippocampal subfield volumes may be more sensitive indicators of cognitive decline^59^ and neuropathological vulnerability^60^ than total hippocampal volume.

Our study has several limitations. First, this study is cross-sectional in nature. All HOSP participants completed MRI scans on average 19 months after their COVID-19 hospitalization. Without pre-infection scans, we could not determine whether the observed measures occurred after COVID-19 hospitalization. In the allotted time frame across four study sites, only four participants who were hospitalized while testing negative for COVID-19 completed 7T MRI. Therefore, the sample size was insufficient to make meaningful comparisons between hospitalization with and without COVID-19 infection. Longitudinal studies and the inclusion of other illnesses, such as pneumonia and sepsis, are needed to determine whether the observed differences resolve over time and whether the effects of COVID-19 hospitalization are primarily due to COVID-19 infection itself or the combined impact of COVID-19 and comorbidities. In fact, studies have compared COVID-19 hospitalization and non-COVID-19 hospitalization for critical illness such as pneumonia, myocardial infarction and other ICU-requiring conditions and found comparable outcomes on brain health and cognitive impairment.^61^ Longitudinal studies show that COVID-19 patients experience a more dramatic decrease in parahippocampal gyrus tissue integrity and orbitofrontal cortex cortical thickness as well as a greater reduction in global brain volume and a greater cognitive decline between baseline and 5-month follow-up compared to controls.^6^ The adverse effect of COVID-19 on brain function and structure appears to fade over time, especially between ICU and non-ICU patients.^62^ Our HOSP and NO HOSP groups were not sex matched. Studies have shown that COVID-19 mortality rate is higher in males^63^ but females are at a higher risk for long COVID-19.^64^ We therefore used multiple linear regression to account for volume differences in neuroimages between males and females. Second, the timing and effect of treatments are not considered in the analysis, although the benefits of hydrocortisone, benzodiazepine, anticoagulant heparin, antiplatelet agents and IL6 receptor antagonists for the treatment of critically ill patients with COVID-19 have been reported. The timing of the COVID-19 vaccines is also not considered. Participants could have received vaccines before COVID-19 infection impacting the presentation of symptom and risk for hospitalization. The third limitation is the analysis of total WMH volume rather than regional WMH volumes. Some studies found a higher WMH volume in COVID-19 patients in the right hemisphere,^65^ especially the right frontal lobe.^8^

In conclusion, we found comparable WMH burden but lower total hippocampal volume with reduced cognitive and memory performance in individuals hospitalized for COVID-19 compared to age-matched non-hospitalized participants. We also demonstrated more associations between hippocampal subfield volumes and age, cognitive performance and plasma biomarkers in the hospitalized group, possibly reflecting lower variance within this group. In the hospitalized group, specific hippocampal subfields such as CA2 + CA3 + DG, SUB and ERC show distinct associations with age, cognitive performance and plasma pTau181.

## Supplementary Material

fcag045_Supplementary_Data

## Data Availability

All data generated and analysed during this study are included in this article. The anonymized raw and processed MRI scans, along with group-level plasma biomarkers and cognitive data, are available upon reasonable request. All GitHub codes used in this work are cited in the methods section.

## Appendix I

7T MRI COVID Consortium collaborators

Sudha Seshadri, MD; Farhaan S. Vahidy, PhD; Timothy D. Girard, MD; Heidi I. L. Jacobs, PhD; Akram A. Hosseini, MD, PhD; and Tamer S. Ibrahim, PhD.
