# Supplementary material for: Examining neuroimaging biomarkers, plasma biomarkers and cognitive functions in patients with recovered COVID-19 infection: a multicentre study using 7T MRI
Source: Brain Commun. 2026 Mar 9;8(2):fcag045. doi: 10.1093/braincomms/fcag045 (PMC12967851; doi:10.1093/braincomms/fcag045)
Supplement: fcag045_Supplementary_Data [file fcag045_supplementary_data.docx]

**Supplementary material**

**
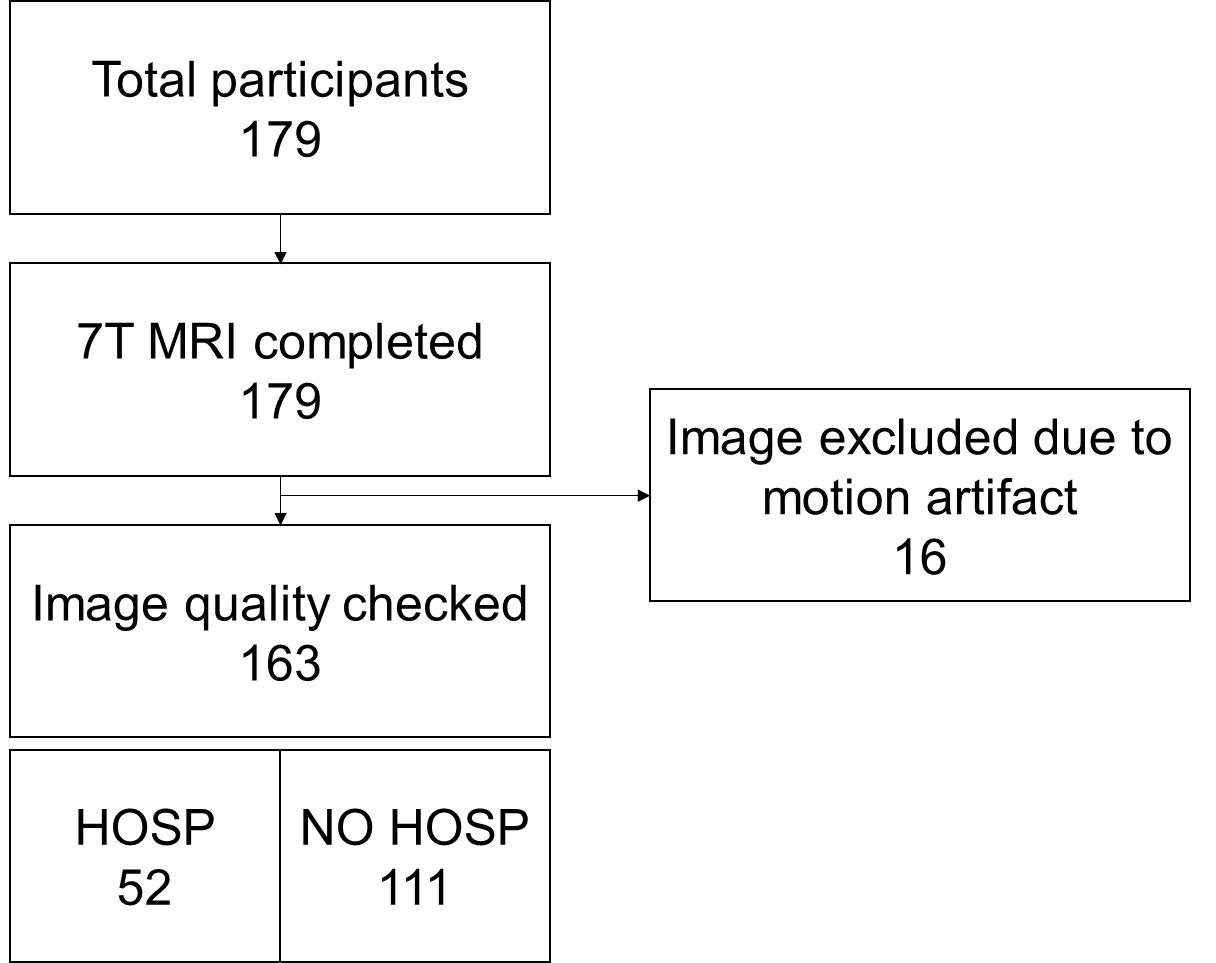
**

Supplementary Figure 1. Study design. A total of 179 participants, including those with COVID-19 hospitalization (HOSP) and those without hospitalization (NO HOSP), consented and completed 7T MRI scans. Cognitive assessments, blood collections, and neuroimaging were conducted across the study sites. After image quality control, 16 images with motion artifacts were excluded, resulting in a total of 163 participants (52 HOSP and 111 NO HOSP).


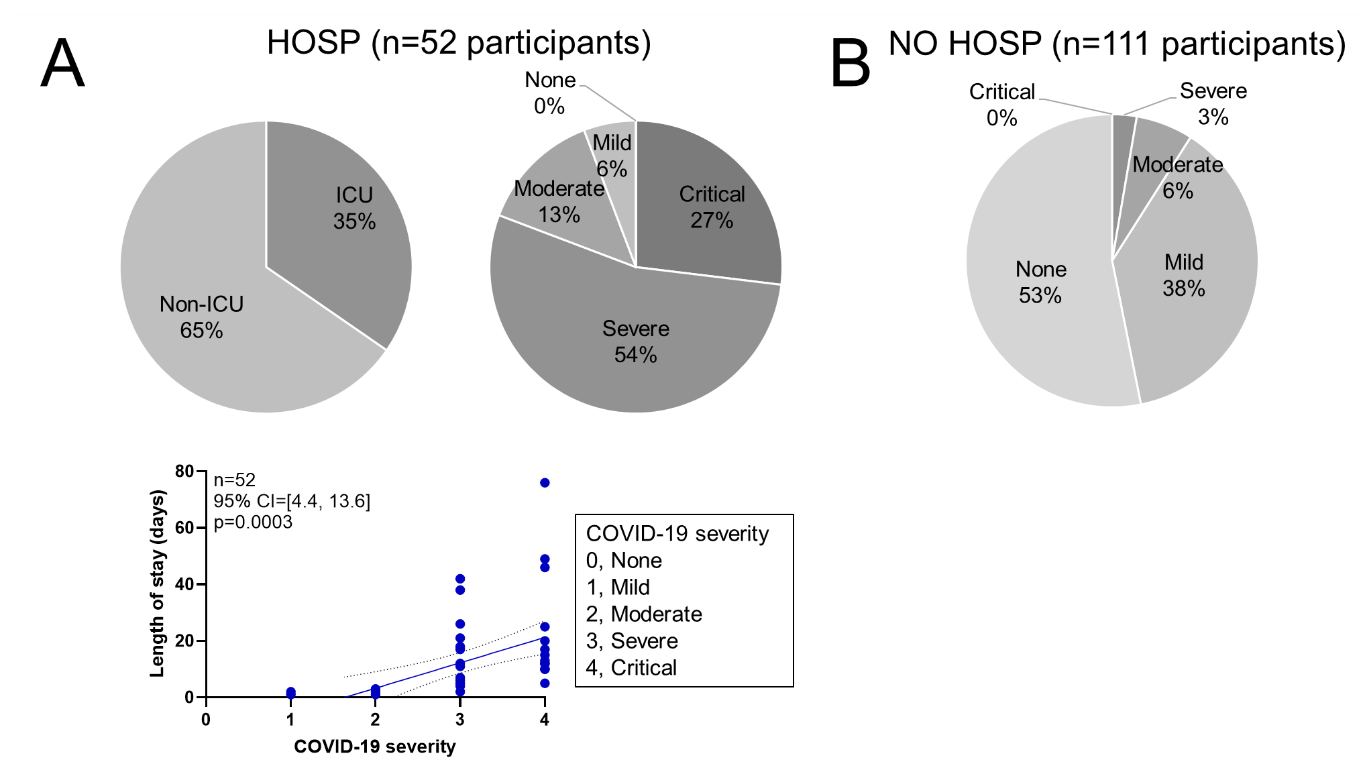


Supplementary Figure 2. COVID-19 severity in HOSP and NO HOSP groups. (A) In the HOSP group, 35% of the 52 participants were admitted to the intensive care unit (ICU). Based on the WHO classification of COVID-19 severity, 27% were classified as critical, 54% as severe, 13% as moderate, and the remaining 6% had mild symptoms. COVID-19 severity was significantly associated with the length of hospitalization (n=52 participants, p=0.0003). Each datapoint represents a participant in HOSP. Linear regression is performed and the p-value is reported. (B) In contrast, in the NO HOSP group, only 3% and 6% of the 111 participants had severe and moderate symptoms, respectively, while the remaining participants experienced mild to no symptoms.


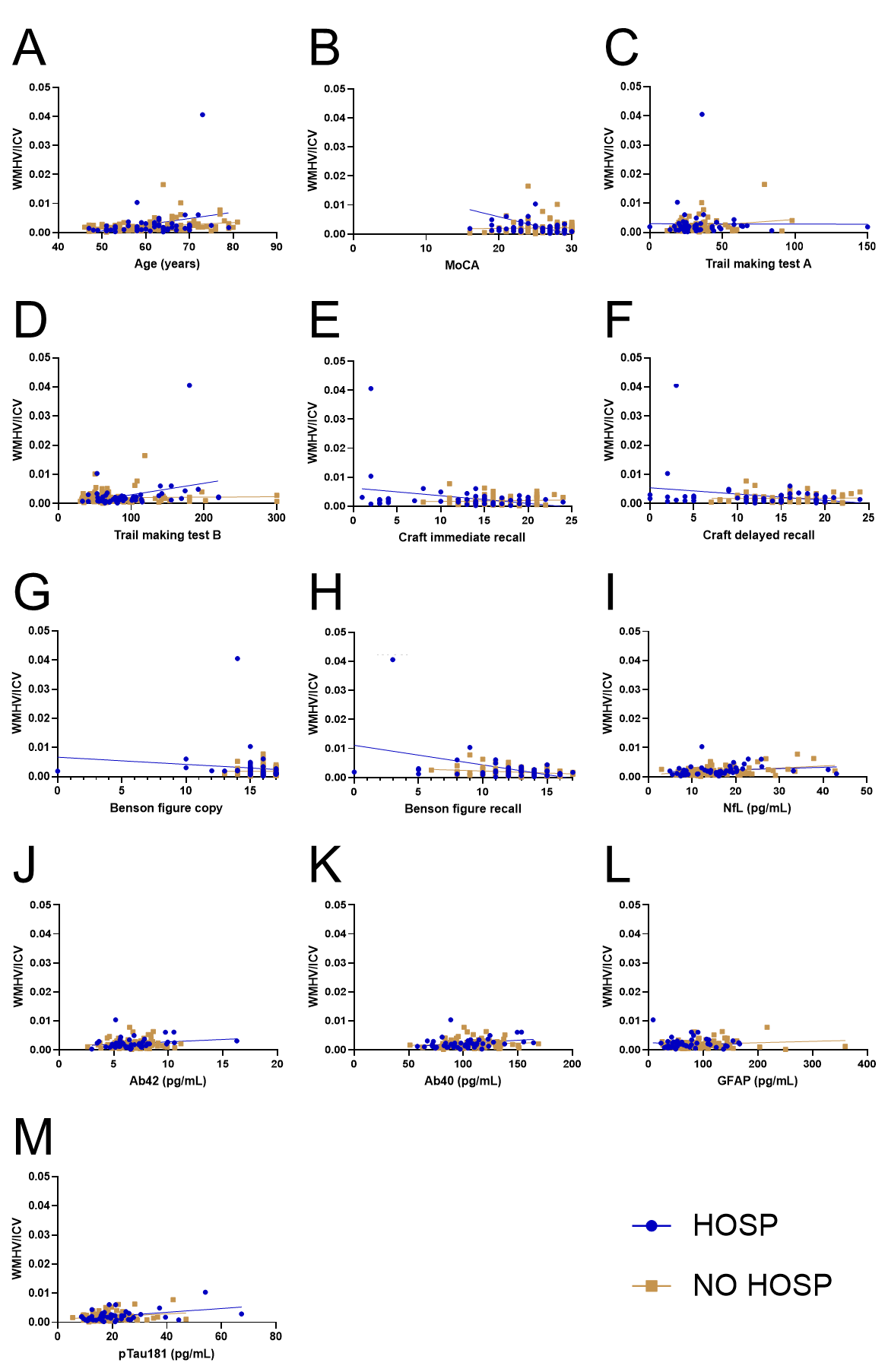


Supplementary Figure 3. Association of WMH burden with (A) age, (B-H) cognitive performance, and (I-M) plasma biomarkers. Each datapoint represents a participant in each group where circles are in HOSP and squares are in NO HOSP groups. Normality tests confirmed a non-Gaussian distribution; non-parametric Spearman correlation was performed; the resulting z-score from Fisher’s r to z transformation testing the difference between two correlation coefficients was reported in Table 3.


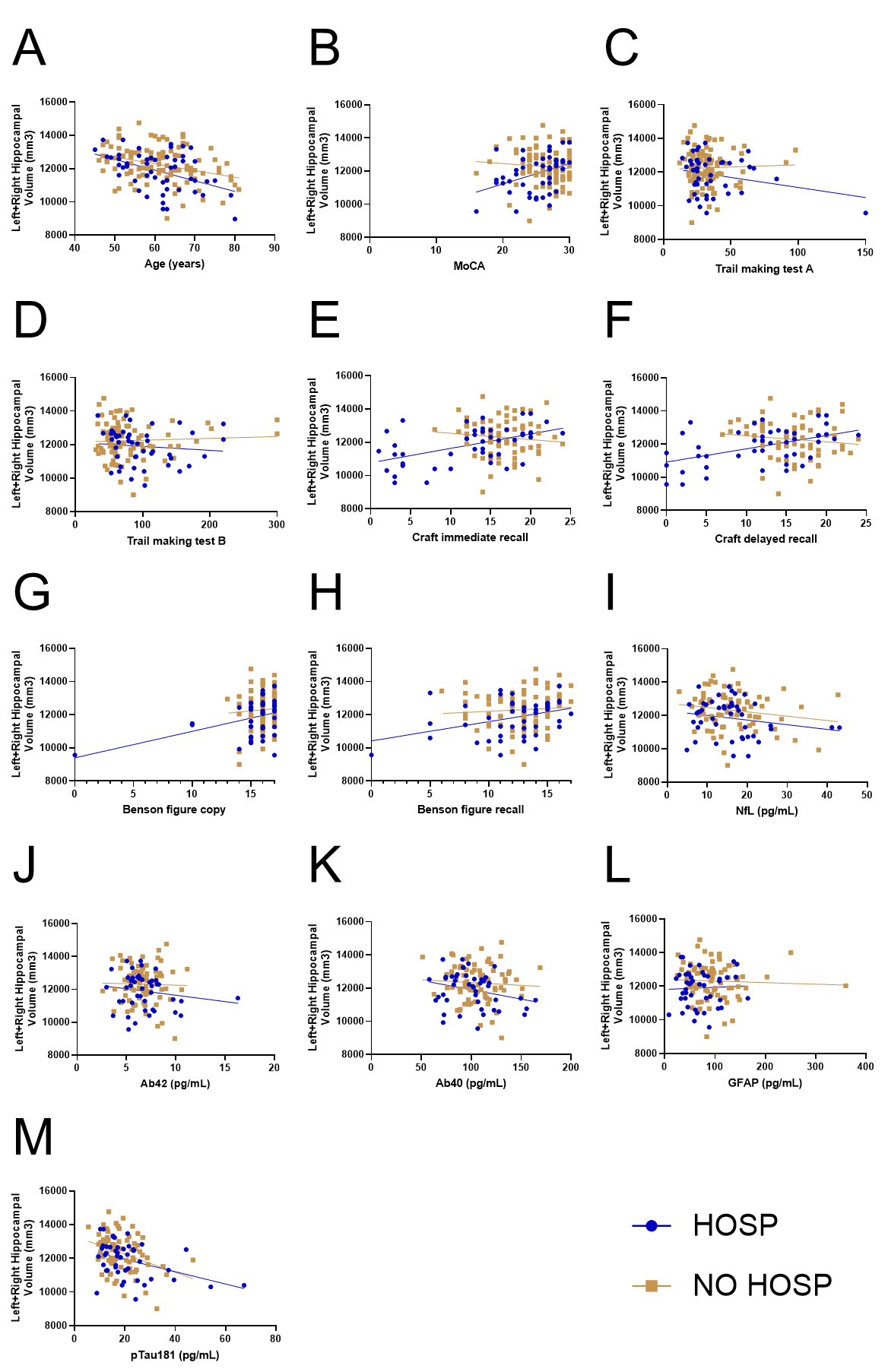


Supplementary Figure 4. Association of total hippocampal volume with (A) age, (B-H) cognitive performance, and (I-M) plasma biomarkers. Each datapoint represents a participant in each group where circles are in HOSP and squares are in NO HOSP groups. Normality tests confirmed a Gaussian distribution; Pearson correlation was performed; the resulting z-score from Fisher’s r to z transformation testing the difference between two correlation coefficients was reported in Table 4.

Supplementary Table 1. WHO classification of COVID-19 severity. SpO2, oxygen saturation rate indicating the oxygen amount being carried by red blood cells in the body which should be 95% or above; RR, respiratory rate which should be about 20 breaths per minute normally; ARDS, acute respiratory distress syndrome.

|  | Based on available clinical records | Based on self-report, if clinical records are not available |
| --- | --- | --- |
| Mild | No hypoxia or pneumonia | Did not receive oxygen |
| Moderate | Clinical signs of non-severe pneumonia AND SpO2≥90% on room air | Did not receive oxygen |
| Severe | Clinical signs of severe pneumonia AND SpO2≤90% on room air; OR RR>30 breaths/min | Received oxygen (or told us they needed it, but it was not available) |
| Critical | ARDS; OR sepsis/septic shock; OR pulmonary embolism, acute coronary syndrome, acute stroke | Received invasive ventilation (or max available respiratory support) |

Supplementary Table 2. Study sites, MR scanners, head coils, and sample sizes in HOSP and NO HOSP groups. 7T MRI data was acquired using different head coils and three 7T scanners: one Philips Achieva and two Siemens Magnetom systems.

| Study Site | Head Coil, MR Scanner, Imaging Location | Sample Size  HOSP/ NO HOSP |
| --- | --- | --- |
| University of Nottingham | Nova Medical, 7T Philips Achieva, Nottingham UK | 8/ 32 |
| Houston Methodist Research Institute | Nova Medical, 7T Siemens Magnetom, Houston USA | 17/ 23 |
| University of Texas Health Science Center at San Antonio |  | 5/ 33 |
| University of Pittsburgh | Tic-Tac-Toe, 7T Siemens Magnetom, Pittsburgh USA | 22/ 23 |
|  |  | 52/ 111 |

Supplementary Table 3. 7T MRI sequence parameters. T1-MP2RAGE, T2-FLAIR, and T2-TSE images were acquried and analyzed to quantify the intracranial volume, white matter hyperintensity volume, and hippocampal subfield volume.

|  | T1-MP2RAGE | T2-FLAIR | T2-TSE |
| --- | --- | --- | --- |
| Resolution (mm) | 0.55 x 0.55 x 0.55 | 0.75 x 0.75 x 1.5 | 0.375 x 0.375 x 1.5 |
| Echo Time (ms) | 2.22 | 99 | 61 |
| Inversion Time (ms) | 800 & 2500 | 2900 | - |
| Repetition Time (ms) | 6000 | 14000 | 10060 |
| Acceleration Factor | 2 | 2 | 2 |
| Acquisition Time (min) | 12:44 | 11:28 | 3:53 |

Supplementary Table 4. Raw data for WMH burden, total hippocampal volume, age, cognitive performance, and plasma biomarkers in 163 subjects. Blank cells indicate data points that were either originally missing or did not pass quality control.

| **Subject ID #** | **Group** | **WMHV/ICV** | **Total hippocampal volume (mm3)** | **Age (years)** | **MoCA** | **Trail making test A** | **Trail making test B** | **Craft immediate recall** | **Craft delayed recall** | **Benson figure copy** | **Benson figure recall** | **NfL (pg/mL)** | **Ab42 (pg/mL)** | **Ab40 (pg/mL)** | **GFAP (pg/mL)** | **pTau181 (pg/mL)** |
| --- | --- | --- | --- | --- | --- | --- | --- | --- | --- | --- | --- | --- | --- | --- | --- | --- |
| **1** | HOSP | 0.00095 | 12829.01 | 51 | 27 | 14 | 54 | 14 | 14 | 17 | 16 | 11.18904 | 6.238165 | 86.06504 | 75.14796 | 26.76714 |
| **2** | HOSP | 0.001444 | 13728.18 | 47 | 29 | 31 | 75 | 20 | 19 | 17 | 16 | 15.48676 | 5.040883 | 71.51445 | 35.88225 | 11.50999 |
| **3** | HOSP | 0.000837 | 11280.25 | 70 | 26 | 25 | 62 | 16 | 15 | 16 | 14 | 7.648925 | 4.665069 | 75.51806 | 41.04907 | 13.04464 |
| **4** | HOSP | 0.001422 | 13265.98 | 69 | 23 | 22 | 114 | 12 | 11 | 16 | 12 | 19.34193 | 7.964122 | 91.84558 | 68.63354 | 15.57274 |
| **5** | HOSP | 0.001297 | 12119.46 | 65 | 27 | 22 | 84 | 16 | 15 | 16 | 14 | 15.88569 | 5.004009 | 101.5655 | 69.17799 | 9.456243 |
| **6** | HOSP | 0.000867 | 12750.89 | 67 | 26 | 34 | 71 | 17 | 15 | 16 | 16 | 16.07507 | 6.111686 | 94.86937 | 45.7082 | 11.25748 |
| **7** | HOSP | 0.002101 | 12306.75 | 53 | 28 | 17 | 54 | 20 | 21 | 17 | 15 | 8.629229 | 8.09653 | 117.9945 | 55.70196 | 17.00148 |
| **8** | HOSP | 0.000233 | 13479.26 | 64 | 27 | 26 | 81 | 14 | 12 | 16 | 11 | 16.12755 | 6.539086 | 97.12701 | 136.8902 | 21.03331 |
| **9** | HOSP | 0.001783 | 12058.64 | 64 | 22 | 25 | 92 | 14 | 13 | 16 | 17 | 18.64866 | 6.610645 | 102.1702 | 78.36776 | 18.28168 |
| **10** | HOSP | 0.000288 | 12139.56 | 53 | 29 | 24 | 64 | 18 | 19 | 16 | 14 | 7.403231 | 2.982375 | 117.0831 | 49.60593 | 16.91936 |
| **11** | HOSP | 0.001142 | 11608.8 | 51 | 20 | 26 | 101 | 14 | 12 | 15 | 16 | 6.531306 | 6.114708 | 71.95749 | 42.39102 | 11.45916 |
| **12** | HOSP | 0.000853 | 12721.71 | 48 | 23 | 25 | 56 | 15 | 13 | 15 | 12 | 9.079587 | 5.149061 | 84.91602 | 48.28091 | 21.54836 |
| **13** | HOSP | 0.004925 | 11296.45 | 63 | 19 | 32 | 192 | 10 | 9 | 15 | 11 | 19.4794 | 6.861136 | 124.532 | 82.28279 | 37.32839 |
| **14** | HOSP | 0.000835 | 13736.27 | 52 | 30 | 18 | 33 | 19 | 20 | 17 | 16 | 7.875835 | 6.438653 | 91.18215 | 32.56948 | 10.21662 |
| **15** | HOSP | 0.000574 | 12592.13 | 51 | 28 | 44 | 80 | 20 | 20 | 16 | 15 | 16.26534 | 6.602742 | 74.37727 | 85.73783 | 10.87269 |
| **16** | HOSP | 0.003702 | 10677.32 | 56 | 24 | 23 | 67 | 19 | 17 | 15 | 13 | 18.68234 | 7.815796 | 122.7224 | 106.1684 | 24.99773 |
| **17** | HOSP | 0.002074 | 12300.14 | 58 | 26 | 64 | 220 | 15 | 16 | 15 | 14 | 17.84338 | 8.276573 | 108.9626 | 108.5834 | 12.45265 |
| **18** | HOSP | 0.002743 | 10766.52 | 63 | 28 | 58 | 138 | 15 | 15 | 17 | 14 | 21.58179 | 7.669294 | 155.3473 | 45.57899 | 30.41837 |
| **19** | HOSP | 0.002323 | 12226.19 | 51 | 27 | 67 | 107 | 12 | 11 | 16 | 13 | 8.10883 | 5.433078 | 97.86577 | 43.98602 | 20.95962 |
| **20** | HOSP | 0.002331 | 13239.78 | 56 | 27 | 63 | 220 | 22 | 20 | 16 | 11 | 12.86616 | 3.487892 | 92.1441 | 50.48398 | 15.88655 |
| **21** | HOSP | 0.006107 | 10400.72 | 72 | 24 | 37 | 155 | 8 |  | 16 | 11 | 22.85986 | 10.51356 | 153.4043 | 78.34304 | 18.86315 |
| **22** | HOSP | 0.003431 | 12651.46 | 59 | 29 | 25 | 53 | 14 | 16 | 16 | 12 | 9.705487 | 6.442955 | 114.548 | 78.66998 | 17.07889 |
| **23** | HOSP | 0.001786 | 10716 | 66 | 21 | 48 | 169 | 4 | 0 | 16 | 11 | 20.15682 | 6.899902 | 110.33 | 112.475 | 39.50836 |
| **24** | HOSP | 0.004396 | 12701.65 | 63 | 23 | 58 | 174 | 13 | 9 | 15 | 15 | 21.93422 | 5.643274 | 80.60454 | 42.19881 | 12.57641 |
| **25** | HOSP | 0.001245 | 13318.69 | 64 | 19 | 25 | 155 | 4 | 3 | 16 | 5 | 16.92905 | 5.587686 | 123.2943 | 143.752 | 15.29178 |
| **26** | HOSP | 0.00118 | 12529.64 | 60 | 29 | 49 | 106 | 20 | 19 | 17 | 8 | 17.44394 | 5.487919 | 57.9631 | 52.47871 | 23.3266 |
| **27** | HOSP | 0.002633 | 10598.51 | 70 | 27 | 32 | 87 | 4 | 5 | 15 | 5 | 18.88709 | 9.954302 | 127.7067 | 60.75279 | 19.54006 |
| **28** | HOSP | 0.00143 | 12211.22 | 53 | 23 | 25 | 79 | 12 | 12 | 17 | 12 | 7.81805 | 7.558158 | 82.43338 | 51.95075 | 12.14321 |
| **29** | HOSP | 0.001961 | 9932.494 | 62 | 27 | 27 | 83 | 3 | 5 | 14 | 14 | 4.892094 | 5.88726 | 71.78445 | 58.93497 | 8.812516 |
| **30** | HOSP | 0.003044 | 11472.94 | 60 | 19 | 35 | 113 | 1 | 0 | 10 | 5 | 12.17725 | 16.2833 | 67.09826 | 52.50359 | 16.30083 |
| **31** | HOSP | 0.010352 | 10302.54 | 58 | 25 | 19 | 53 | 2 | 2 | 15 | 9 | 12.22389 | 5.169632 | 88.50541 | 8.291974 | 54.0898 |
| **32** | HOSP | 0.003116 |  | 66 | 28 | 20 | 41 | 20 | 19 | 15 | 12 | 20.0104 |  | 104.3234 | 154.3375 | 26.08992 |
| **33** | HOSP | 0.000769 | 12666.84 | 49 | 28 | 20 | 41 | 2 | 2 | 17 | 15 | 5.49696 | 6.329663 | 74.44409 | 29.09967 | 14.07956 |
| **34** | HOSP | 0.001707 | 10402.28 | 79 | 26 | 32 | 102 | 10 | 12 | 15 | 10 | 16.21486 | 7.320237 | 112.097 | 64.3528 | 27.77521 |
| **35** | HOSP | 0.002332 | 12780.64 | 56 |  |  |  |  |  |  |  |  |  |  |  |  |
| **36** | HOSP | 0.002039 |  | 58 | 28 | 43 | 84 | 18 | 20 | 12 | 12 | 33.16963 | 9.844891 | 137.9432 | 166.595 | 23.46757 |
| **37** | HOSP | 0.0022 | 12437.12 | 70 | 28 | 31 | 78 | 19 |  | 14 | 13 | 14.27499 | 7.350133 | 112.533 | 108.9902 | 16.31514 |
| **38** | HOSP | 0.00101 | 11267.88 | 55 | 20 | 23 | 89 | 3 | 5 | 17 | 16 | 43.07861 | 4.273564 | 64.42362 | 32.33074 | 12.55275 |
| **39** | HOSP | 0.002328 | 12459.78 | 67 | 25 | 21 | 61 | 13 | 12 | 15 | 15 | 12.93554 | 5.816534 | 83.80929 | 22.69316 | 23.45055 |
| **40** | HOSP | 0.001452 | 12551.64 | 58 | 29 | 43 | 60 | 24 | 24 | 17 | 14 | 14.18991 | 8.002882 | 103.0518 | 140.9676 | 19.45011 |
| **41** | HOSP | 0.001 | 11284.91 | 59 | 22 | 35 | 60 | 3 | 4 | 17 | 10 | 41.13944 | 10.55909 | 164.4361 | 164.9437 | 20.15819 |
| **42** | HOSP | 0.006074 | 11810.79 | 69 | 21 | 24 | 140 | 14 | 16 | 10 | 8 | 11.33972 | 7.508664 | 104.4306 | 41.77608 | 25.87399 |
| **43** | HOSP | 0.040553 | 11387.45 | 73 | 16 | 36 | 180 | 2 | 3 | 14 | 3 | 25.83871 | 9.740493 | 149.4797 | 90.34293 | 21.24375 |
| **44** | HOSP | 0.0023 |  | 63 | 22 | 32 | 103 | 3 | 2 | 17 | 11 |  |  |  |  |  |
| **45** | HOSP | 0.002886 | 9564.266 | 62 | 25 | 25 | 65 | 17 | 15 | 15 | 13 | 20.26717 | 5.219526 | 106.4035 | 88.44258 | 24.24751 |
| **46** | HOSP | 0.001925 | 10397.19 | 70 | 20 | 0 | 103 | 14 | 10 | 13 | 13 | 7.015261 | 3.645177 | 72.58093 | 35.33163 | 67.37423 |
| **47** | HOSP | 0.002444 |  | 75 | 27 | 24 | 90 | 4 | 4 | 15 | 12 | 13.48636 | 4.862905 | 88.99417 | 54.85871 | 8.702928 |
| **48** | HOSP | 0.000874 | 12526.11 | 65 | 30 | 26 | 65 | 19 | 19 | 17 | 15 | 15.55924 | 7.568699 | 110.2964 | 123.8096 | 44.31549 |
| **49** | HOSP | 0.000605 | 11593.32 | 58 | 27 | 84 | 115 | 13 | 11 | 15 | 13 | 8.402942 | 6.413421 | 104.29 | 35.87134 | 16.31571 |
| **50** | HOSP | 0.003472 | 11184.87 | 62 | 23 | 46 | 142 | 15 | 18 | 16 | 13 | 25.94905 | 5.580022 | 106.7304 | 110.0524 | 17.18999 |
| **51** | HOSP | 0.000539 |  | 61 | 28 | 32 | 70 | 16 | 15 | 15 | 12 |  |  |  |  |  |
| **52** | HOSP | 0.001904 | 9570.355 | 62 | 16 | 150 |  | 7 | 0 | 0 | 0 | 16.54427 |  |  |  |  |
| **53** | NO HOSP | 0.001465 | 11745.58 | 61 | 30 | 24.89 | 56.94 |  |  |  |  |  |  |  |  |  |
| **54** | NO HOSP | 0.016491 |  | 64 | 24 | 79.06 | 118.69 |  |  |  |  |  |  |  |  |  |
| **55** | NO HOSP | 0.004124 | 10787.95 | 66 | 28 | 21.12 | 60.08 |  |  |  |  |  |  |  |  |  |
| **56** | NO HOSP | 0.001271 | 11628.47 | 64 | 27 | 22.93 | 40.25 |  |  |  |  |  |  |  |  |  |
| **57** | NO HOSP | 0.005542 | 11909.42 | 66 | 27 | 27.101 | 51.501 |  |  |  |  |  |  |  |  |  |
| **58** | NO HOSP | 0.00157 |  | 60 | 30 | 17.18 | 32.32 |  |  |  |  |  |  |  |  |  |
| **59** | NO HOSP | 0.000918 | 11558.05 | 58 | 29 | 28.16 | 54.44 |  |  |  |  |  |  |  |  |  |
| **60** | NO HOSP | 0.001651 | 12664.71 | 59 | 24 | 20.12 | 53.35 |  |  |  |  |  |  |  |  |  |
| **61** | NO HOSP | 0.001077 | 11531.06 | 56 | 27 | 22 | 41 |  |  |  |  |  |  |  |  |  |
| **62** | NO HOSP | 0.002258 | 12273.07 | 61 | 28 | 20.66 | 52.501 |  |  |  |  |  |  |  |  |  |
| **63** | NO HOSP | 0.000812 | 11900.61 | 61 | 28 | 18.66 | 33.81 |  |  |  |  |  |  |  |  |  |
| **64** | NO HOSP | 0.001411 | 12668.9 | 62 | 28 | 28.001 | 63.35 |  |  |  |  |  |  |  |  |  |
| **65** | NO HOSP | 0.000793 | 11698.46 | 69 | 30 | 22.88 | 30.25 |  |  |  |  |  |  |  |  |  |
| **66** | NO HOSP | 0.002222 | 10796.36 | 51 | 28 | 25.06 | 54.12 |  |  |  |  |  |  |  |  |  |
| **67** | NO HOSP | 0.001007 | 13539.69 | 67 | 25 | 59.94 | 71.81 |  |  |  |  |  |  |  |  |  |
| **68** | NO HOSP | 0.00211 |  | 57 | 20 | 31.56 | 63.28 |  |  |  |  |  |  |  |  |  |
| **69** | NO HOSP | 0.001459 | 11926.19 | 61 | 23 | 41.53 | 110.501 |  |  |  |  |  |  |  |  |  |
| **70** | NO HOSP | 0.000761 | 12702.52 | 54 | 28 | 35.69 | 48.28 |  |  |  |  |  |  |  |  |  |
| **71** | NO HOSP | 0.002003 | 14022.35 | 59 | 23 | 18.04 | 66.47 |  |  |  |  |  |  |  |  |  |
| **72** | NO HOSP | 0.010207 | 12160.3 | 68 | 28 | 35.81 | 50.47 |  |  |  |  |  |  |  |  |  |
| **73** | NO HOSP | 0.001323 | 12326.19 | 54 | 24 | 26.22 | 47.84 |  |  |  |  |  |  |  |  |  |
| **74** | NO HOSP | 0.000848 | 11766.41 | 64 | 25 | 39.37 | 100.69 |  |  |  |  |  |  |  |  |  |
| **75** | NO HOSP | 0.001186 | 11908.54 | 62 | 26 | 31.501 | 60.11 |  |  |  |  |  |  |  |  |  |
| **76** | NO HOSP | 0.001362 | 12784.42 | 66 | 26 | 27 | 61 | 15 | 12 | 15 | 11 | 17.8202 | 5.571451 | 111.4268 | 98.15723 | 25.06222 |
| **77** | NO HOSP | 0.00104 | 11887.31 | 62 | 27 | 43 | 141 | 18 | 17 | 16 | 13 | 22.89388 | 7.16074 | 98.86477 | 127.57 | 20.56981 |
| **78** | NO HOSP | 0.00116 |  | 67 | 27 | 29 | 50 | 17 | 18 | 17 | 10 | 12.89391 | 6.265151 | 87.60491 | 122.9652 | 15.16203 |
| **79** | NO HOSP | 0.001773 | 12785.59 | 52 | 25 | 22 | 52 | 18 | 16 | 16 | 17 | 20.01475 | 8.060288 | 109.1625 | 70.71274 | 15.65042 |
| **80** | NO HOSP | 0.002213 | 13229.76 | 51 | 30 | 29 | 46 | 14 | 13 | 16 | 16 | 14.10202 | 6.618761 | 88.79405 | 128.757 | 12.9106 |
| **81** | NO HOSP | 0.001444 | 11303.4 | 63 |  | 24 | 60 | 15 | 17 | 17 | 10 | 13.76862 | 5.314561 | 97.20023 | 115.4769 | 20.70712 |
| **82** | NO HOSP | 0.001306 | 14073.36 | 67 | 27 | 32 | 84 | 17 | 19 | 16 | 14 | 10.90358 | 4.385459 | 80.38219 | 79.74376 | 16.52983 |
| **83** | NO HOSP | 0.00109 | 13905.54 | 62 | 29 | 33 | 53 | 20 | 19 | 16 | 14 | 9.394167 | 5.336352 | 97.65977 | 92.32916 | 23.02568 |
| **84** | NO HOSP | 0.000761 | 13478.37 | 68 | 19 | 32 | 300 | 12 | 9 | 15 | 13 | 18.02163 | 7.029235 | 119.7084 | 94.91521 | 25.11859 |
| **85** | NO HOSP | 0.001848 | 11188.18 | 73 | 22 | 54 | 146 | 13 | 13 | 16 | 15 | 18.50301 | 4.463015 | 103.9955 | 148.7408 | 24.08961 |
| **86** | NO HOSP | 0.002202 | 12188.7 | 63 | 24 | 33 | 180 | 15 | 12 | 17 | 12 | 14.94936 | 6.979711 | 106.7167 | 111.2211 | 26.93448 |
| **87** | NO HOSP | 0.000983 | 12552.68 | 59 | 29 | 40 | 58 | 15 | 15 | 16 | 16 | 23.71154 | 5.17737 | 112.3631 | 203.2956 | 24.16589 |
| **88** | NO HOSP | 0.00079 | 11626.68 | 57 | 28 | 37 | 100 | 19 | 19 | 16 | 14 | 11.9456 | 4.908395 | 62.7588 | 55.71311 | 29.20418 |
| **89** | NO HOSP | 0.002187 | 12043.56 | 57 | 26 | 36 | 72 | 15 | 15 | 14 | 13 | 12.82486 | 5.565141 | 81.24607 | 72.00531 | 17.60983 |
| **90** | NO HOSP | 0.001556 | 11045.59 | 65 | 27 | 34 | 78 | 14 | 14 | 16 | 9 | 11.22213 | 5.905464 | 116.6637 | 66.20283 | 14.0577 |
| **91** | NO HOSP | 0.001528 | 13764.41 | 61 | 30 | 15 | 39 | 16 | 16 | 16 | 17 | 15.78195 | 8.489003 | 117.6258 | 108.021 | 12.77289 |
| **92** | NO HOSP | 0.000165 | 13998.75 | 51 | 25 | 34 | 61 | 19 | 22 | 15 | 13 | 11.98922 | 6.638325 | 81.82651 | 250.6298 | 11.42799 |
| **93** | NO HOSP | 0.000378 | 11877.86 | 47 | 16 | 40 | 66 | 16 | 15 | 17 | 15 | 11.27325 | 7.444287 | 132.7693 | 62.15106 | 15.92027 |
| **94** | NO HOSP | 0.000771 |  | 57 | 21 | 24 | 180 | 12 | 16 | 17 | 13 | 10.87661 | 5.814705 | 76.47841 | 41.37248 | 11.94564 |
| **95** | NO HOSP | 0.001006 | 12947.07 | 55 | 26 | 34 | 202 | 21 | 21 | 13 | 10 | 10.60443 | 6.514508 | 80.41487 | 96.25827 | 10.35064 |
| **96** | NO HOSP | 0.004042 | 13298.55 | 66 | 22 | 98 | 197 | 15 | 11 | 17 | 8 | 19.55929 | 4.402762 | 83.41834 | 139.5458 | 21.61329 |
| **97** | NO HOSP | 0.000718 | 12836.37 | 54 | 24 | 32 | 88 | 19 | 18 | 15 | 15 | 8.649163 | 5.29042 | 75.40674 | 47.36224 | 9.468683 |
| **98** | NO HOSP | 0.000607 | 12013.53 | 58 | 26 | 59 | 90 | 15 | 14 | 17 | 15 | 13.51471 | 3.82481 | 76.0582 | 54.44169 | 16.69486 |
| **99** | NO HOSP | 0.001922 | 12000.29 | 50 | 26 |  |  | 17 | 16 | 17 | 15 | 7.881773 | 8.569961 | 123.6729 | 66.33722 | 14.45468 |
| **100** | NO HOSP | 0.003473 | 11471.62 | 63 | 29 | 20 | 34 | 21 | 23 | 17 | 10 | 18.10421 | 6.315552 | 114.0018 | 105.0924 | 18.46107 |
| **101** | NO HOSP | 0.002557 | 13432.59 | 47 | 25 | 52 | 144 | 18 | 12 | 15 | 6 | 2.983347 | 6.214439 | 103.2309 | 36.69402 | 9.620074 |
| **102** | NO HOSP | 0.002865 | 12645.42 | 50 | 24 | 32 | 300 | 11 | 10 | 14 | 10 | 9.162717 | 6.97175 | 110.2874 | 24.99683 | 13.3592 |
| **103** | NO HOSP | 0.001654 | 13870.72 | 51 | 29 |  |  |  |  |  |  | 10.34265 | 3.571536 | 51.21907 | 26.33723 | 5.480536 |
| **104** | NO HOSP | 0.003444 | 10723.43 | 55 | 28 | 37 | 44 | 18 | 19 | 17 | 15 | 13.49886 | 5.590512 | 88.90891 | 54.4089 | 12.85734 |
| **105** | NO HOSP | 0.001132 |  | 59 | 28 | 17 | 36 | 18 | 17 | 17 | 17 | 16.64711 | 5.823611 | 109.7112 | 94.0863 | 18.4204 |
| **106** | NO HOSP | 0.0025 | 11947.34 | 68 | 24 | 23 | 60 | 17 | 18 | 16 | 14 | 10.98861 | 5.728776 | 88.58683 | 51.10395 | 9.291324 |
| **107** | NO HOSP | 0.004574 | 13760.27 | 63 |  |  |  |  |  |  |  | 14.26263 | 4.606222 | 95.34969 |  | 17.69466 |
| **108** | NO HOSP | 0.001362 | 12397.43 | 57 | 26 | 40 | 94 | 16 | 17 | 15 | 13 | 8.865247 | 6.344679 | 95.48886 | 43.59902 | 10.27137 |
| **109** | NO HOSP | 0.002482 | 12398.8 | 48 | 26 | 22 | 48 | 15 | 15 | 17 | 15 | 19.22882 | 8.021844 | 134.3174 | 143.3414 | 25.94354 |
| **110** | NO HOSP | 0.002497 | 11688.46 | 53 | 29 | 34 | 82 | 20 | 22 | 17 | 13 | 11.96342 | 6.928405 | 113.7442 | 85.17273 | 14.91987 |
| **111** | NO HOSP | 0.00182 | 12508.5 | 46 | 29 | 34 | 48 | 12 | 11 | 17 | 12 | 33.55547 | 6.243471 | 145.2713 | 126.2044 | 22.60048 |
| **112** | NO HOSP | 0.002533 | 10647.43 | 50 |  |  |  |  |  |  |  | 11.56673 | 5.100962 | 87.93517 | 69.85351 | 16.70635 |
| **113** | NO HOSP | 0.002965 | 10112.59 | 72 |  |  |  |  |  |  |  |  |  |  |  |  |
| **114** | NO HOSP | 0.00186 | 11729.91 | 72 |  |  |  |  |  |  |  |  |  |  |  |  |
| **115** | NO HOSP | 0.003678 | 12882.59 | 61 |  |  |  |  |  |  |  | 17.87584 | 8.43252 | 137.1787 | 91.23856 | 23.73957 |
| **116** | NO HOSP | 0.002048 | 13239.05 | 74 | 27 | 21 | 33 | 17 | 12 | 17 | 13 | 42.71554 | 11.15557 | 168.9462 | 127.1709 | 27.09598 |
| **117** | NO HOSP | 0.002802 | 12820.03 | 65 |  |  |  |  |  |  |  | 20.05676 | 8.559195 | 130.9934 | 111.8704 | 15.09933 |
| **118** | NO HOSP | 0.00619 | 11174.04 | 68 |  |  |  |  |  |  |  | 26.89015 | 6.762601 | 103.7688 | 83.88165 | 22.19599 |
| **119** | NO HOSP | 0.00515 | 12988.09 | 62 | 24 | 22 | 60 | 16 | 16 | 16 | 11 | 25.01922 | 8.408657 | 138.1398 | 119.6441 | 17.62261 |
| **120** | NO HOSP | 0.006251 | 9939.184 | 77 | 21 | 34 | 105 | 15 | 12 | 16 | 10 | 37.84614 | 8.628022 | 121.0967 | 134.6797 | 28.23258 |
| **121** | NO HOSP | 0.005236 | 9764.208 | 67 | 27 | 40 | 67 | 21 | 18 | 14 | 12 | 14.20671 | 8.157647 | 109.1866 | 118.892 | 19.69558 |
| **122** | NO HOSP | 0.001919 | 13919.11 | 67 | 30 | 38 | 76 | 18 | 18 | 17 | 15 | 17.70749 | 8.184882 | 129.4115 | 108.6191 | 17.27285 |
| **123** | NO HOSP | 0.000446 | 13613.1 | 48 |  | 23 | 58 | 17 | 17 | 17 | 15 | 29.10761 | 5.508229 | 116.4055 | 109.0222 | 13.20326 |
| **124** | NO HOSP | 0.000709 | 11979.38 | 56 | 28 | 32 | 55 | 17 | 18 | 17 | 14 | 20.5824 | 10.59584 | 151.048 | 146.0765 | 16.31984 |
| **125** | NO HOSP | 0.002355 | 14760.43 | 56 | 26 | 23 | 42 | 14 | 13 | 15 | 14 | 16.3923 | 9.062789 | 130.2623 | 69.62446 | 13.57877 |
| **126** | NO HOSP | 0.002425 | 12768.71 | 62 | 30 | 42 | 67 | 17 | 15 | 17 | 13 | 10.55423 | 7.015926 | 94.4324 | 88.42436 | 10.56451 |
| **127** | NO HOSP | 0.002057 | 13063.23 | 53 | 30 | 19 | 64 | 12 | 10 | 17 | 16 | 9.047168 | 6.31935 | 108.4695 | 80.68165 | 13.35205 |
| **128** | NO HOSP | 0.0029 | 11866.56 | 54 |  |  |  |  |  |  |  |  |  |  |  |  |
| **129** | NO HOSP | 0.00097 | 11176.41 |  |  |  |  |  |  |  |  |  |  |  |  |  |
| **130** | NO HOSP | 0.001195 | 13955.03 |  | 21 | 43 |  | 11 | 12 | 17 | 8 |  |  |  |  |  |
| **131** | NO HOSP | 0.000771 | 13104.75 | 56 | 28 | 58 | 76 | 20 | 17 | 17 | 9 | 13.95649 | 7.977195 | 95.28574 | 60.32647 | 11.40131 |
| **132** | NO HOSP | 0.001148 | 13329.44 | 49 | 24 | 35 | 63 | 12 | 8 | 17 | 12 | 9.079175 | 6.699628 | 89.38997 | 51.2868 | 15.12415 |
| **133** | NO HOSP | 0.000803 | 11879.57 | 69 | 23 | 32 | 115 | 16 | 14 | 17 | 15 | 11.23116 | 6.747099 | 102.2812 | 100.6715 | 13.53094 |
| **134** | NO HOSP | 0.00176 | 11028.56 | 58 | 29 | 29 | 56 | 18 | 18 | 16 | 12 | 6.792072 | 6.889981 | 93.47124 |  | 10.77704 |
| **135** | NO HOSP | 0.000734 | 12992.06 | 54 | 26 | 31 | 62 | 19 | 17 | 15 | 15 | 6.936168 | 5.743793 | 80.36834 | 33.55619 | 12.61663 |
| **136** | NO HOSP | 0.003066 | 11893.83 | 70 | 30 | 32 | 52 | 24 | 22 | 16 | 11 | 23.04581 | 7.485571 | 124.0489 | 161.5044 | 23.47696 |
| **137** | NO HOSP | 0.001189 | 12030.74 | 76 | 27 | 24 | 92 | 15 | 15 | 15 | 10 | 17.43999 | 4.921753 | 97.04467 | 359.7422 | 28.86055 |
| **138** | NO HOSP | 0.001791 | 11024.41 | 80 | 25 | 36 | 133 | 15 | 15 | 15 | 14 | 22.06401 | 5.736113 | 97.90565 | 123.3692 | 36.5989 |
| **139** | NO HOSP | 0.002425 | 10664.73 | 71 | 30 | 29 | 69 | 18 | 19 | 16 | 12 | 27.44065 | 3.72235 | 86.06632 | 88.49732 | 24.30324 |
| **140** | NO HOSP | 0.000636 | 12544.73 | 48 | 27 | 12 | 49 | 16 | 16 | 16 | 12 | 6.158873 | 5.302338 | 84.78788 | 47.01549 | 17.0545 |
| **141** | NO HOSP | 0.002076 | 11785.95 | 76 | 26 | 30 | 86 | 16 | 14 | 16 | 8 | 28.58219 | 6.989994 | 106.0015 | 98.24231 | 21.66335 |
| **142** | NO HOSP | 0.000536 | 12534.95 | 75 | 23 | 35 | 98 | 21 | 20 | 16 | 13 | 20.96368 | 7.067406 | 133.9986 | 94.9562 | 17.40316 |
| **143** | NO HOSP | 0.000523 | 12573.57 | 61 | 18 | 91 | 138 | 14 | 7 | 15 | 13 | 15.27388 | 4.842368 | 115.5362 | 154.833 | 18.46768 |
| **144** | NO HOSP | 0.004029 | 12310.22 | 78 | 30 | 25 | 50 | 23 | 24 | 16 | 15 | 17.67361 | 5.435581 | 93.72347 | 92.31266 | 12.88676 |
| **145** | NO HOSP | 0.002664 | 11768.06 | 54 | 30 | 19 | 38 | 21 | 18 | 17 | 13 | 9.786432 | 7.190303 | 89.01533 | 81.5543 | 14.49399 |
| **146** | NO HOSP | 0.001468 | 11518 | 79 | 30 | 33 | 59 | 19 | 21 | 16 | 14 | 21.44966 | 8.39561 | 150.4252 | 92.80426 | 35.14112 |
| **147** | NO HOSP | 0.000402 | 14386.77 | 51 | 29 | 20 | 35 | 22 | 22 | 17 | 16 | 11.4592 | 8.26716 | 110.295 | 65.38304 | 19.27981 |
| **148** | NO HOSP | 0.001061 | 11903.82 | 65 | 25 | 35 | 153 | 12 | 12 | 15 | 11 | 22.02532 | 2.603289 | 109.7987 | 133.0697 | 47.0554 |
| **149** | NO HOSP | 0.002547 | 10504 | 65 | 28 | 21 | 47 | 18 | 16 | 17 | 15 | 31.95218 | 6.96232 | 125.1194 | 66.30199 | 15.94282 |
| **150** | NO HOSP | 0.004022 | 12508.13 | 71 | 30 | 46 | 69 | 21 | 21 | 16 | 12 | 14.52477 | 7.777585 | 116.7742 | 143.9582 | 15.07901 |
| **151** | NO HOSP | 0.001065 | 13063.25 | 64 | 28 | 26 | 109 | 12 | 12 | 16 | 14 | 14.445 | 9.703808 | 122.0189 | 93.85697 | 20.84938 |
| **152** | NO HOSP | 0.001711 |  | 62 | 29 | 28 | 81 | 16 | 15 | 16 | 12 | 20.376 |  | 134.7768 | 32.62847 | 20.97902 |
| **153** | NO HOSP | 0.000543 | 12973.21 | 59 | 25 | 30 | 55 | 12 | 12 | 16 | 12 | 15.84533 | 7.069115 | 100.9088 | 58.07477 | 11.56477 |
| **154** | NO HOSP | 0.001397 | 12769.27 | 47 | 26 | 16 | 41 | 8 | 8 | 17 | 8 | 8.415458 | 6.588756 | 92.43578 | 64.2657 | 10.72899 |
| **155** | NO HOSP | 0.000818 | 12088.91 | 59 | 28 | 21 | 68 | 17 | 14 | 17 | 13 | 9.047598 | 6.275007 | 93.73668 | 64.07332 | 11.20514 |
| **156** | NO HOSP | 0.000485 | 11066.78 | 54 | 26 | 57 | 101 | 16 | 14 | 16 | 13 | 10.04926 | 8.018956 | 99.57569 | 72.79857 | 14.3301 |
| **157** | NO HOSP | 0.001277 | 13102.92 | 58 | 22 | 28 | 79 | 13 | 12 | 14 | 10 | 22.98441 | 5.882788 | 103.2196 | 72.56916 | 13.09334 |
| **158** | NO HOSP | 0.003614 | 10743.03 | 81 | 29 | 38 | 63 | 16 | 16 | 16 | 13 |  |  |  |  |  |
| **159** | NO HOSP | 0.00305 | 11315.8 | 79 | 28 | 25 | 63 | 21 | 19 | 17 | 13 | 14.08065 | 5.28711 | 118.3176 | 119.3489 | 24.54641 |
| **160** | NO HOSP | 0.000897 | 9001.144 | 63 | 24 | 21 | 86 | 14 | 14 | 14 | 13 | 15.09348 | 9.9089 | 130.318 | 83.36393 | 32.67769 |
| **161** | NO HOSP | 0.001337 | 11388.58 | 70 | 28 | 57 | 82 | 18 | 17 | 16 | 11 | 20.57382 | 9.461806 | 115.7517 | 145.3991 | 21.87085 |
| **162** | NO HOSP | 0.007786 |  | 77 | 26 | 37 | 108 | 11 | 11 | 16 | 9 | 34.12229 | 6.459277 | 100.7105 | 216.7428 | 42.26667 |
| **163** | NO HOSP | 0.001294 | 12035.55 | 69 | 29 | 31 | 98 | 15 | 15 | 15 | 8 | 16.04282 | 9.011109 | 129.6668 | 47.733 | 23.24026 |
